# Fifty Shades of PSMA-Avid Rib Lesions: A Comprehensive Review

**DOI:** 10.3390/cancers17213404

**Published:** 2025-10-22

**Authors:** Amirreza Shamshirgaran, Mohammad Hadi Samadi, Michael Saeed, Sara Harsini, Pegah Sahafi, Ghasemali Divband, Gholamreza Mohammadi, Narjess Ayati, Ramin Sadeghi, Alessio Rizzo, Giorgio Treglia, Emran Askari

**Affiliations:** 1 Urology Research Center, Tehran University of Medical Sciences, Tehran 1416634793, Iran; amirreza.sh.95@gmail.com; 2 Nuclear Medicine Research Center, Mashhad University of Medical Sciences (MUMS), Mashhad 9177948564, Iran; samadimh4011@mums.ac.ir (M.H.S.); michaelsaeed98@gmail.com (M.S.); pegah.sahafi@gmail.com (P.S.); sadeghir@mums.ac.ir (R.S.); askariem@mums.ac.ir (E.A.); 3 Department of Medical Imaging, University of Toronto, Toronto, ON M5S 1A1, Canada; sara.harsini@mail.utoronto.ca; 4 Jam Nuclear Medicine Center, Tehran 1588657915, Iran; divband_ali@yahoo.com; 5 Department of Radiology, Mashhad University of Medical Sciences (MUMS), Mashhad 9177948564, Iran; ghrezamohammadi.95@gmail.com; 6 Department of Theranostics and Nuclear Medicine, St. Vincent’s Hospital, Sydney, NSW 2010, Australia; narjess.ayati@svha.org.au; 7 Department of Nuclear Medicine, Candiolo Cancer Institute, FPO–IRCCS, 10060 Turin, Italy; alessio.rizzo@ircc.it; 8 Clinic of Nuclear Medicine, Imaging Institute of Southern Switzerland, Ente Ospedaliero Cantonale, 6501 Bellinzona, Switzerland; 9 Faculty of Biology and Medicine, University of Lausanne, 1011 Lausanne, Switzerland; 10 Faculty of Biomedical Sciences, Università della Svizzera Italiana, 6900 Lugano, Switzerland

**Keywords:** prostate cancer, PSMA PET/CT, rib metastasis, unspecific bone uptake

## Abstract

**Simple Summary:**

Prostate cancer often spreads to the bones, and the ribs are a frequent site of suspicious findings on advanced scans. New imaging methods are very sensitive, but they sometimes show spots in the ribs that look like cancer even when they are not. This creates uncertainty for doctors and patients, since a mistaken diagnosis of rib metastasis can change treatment decisions and lead to unnecessary therapies. Our review gathers the current knowledge about these rib findings and explains how to tell apart true cancer from harmless changes, such as old fractures or other benign conditions. By highlighting patterns related to scan intensity, lesion number, location, patient risk, and follow-up results, we suggest a practical approach to interpretation. This guidance may help clinicians avoid false alarms, ensure accurate staging, and make treatment more personalized for men with prostate cancer.

**Abstract:**

Background: While prostate-specific membrane antigen (PSMA)-targeted imaging has revolutionized metastatic detection, unspecific bone uptake (UBU)—particularly in the ribs—is a common but diagnostically challenging finding in prostate cancer (PCa) patients. This review aims to synthesize current evidence on PSMA-avid rib lesions in PCa and to propose a structured approach for differentiating true metastases from benign mimics. Methods: A comprehensive literature search across PubMed, EMBASE, Scopus, and Web of Science identified relevant studies on PSMA imaging interpretation, tracer-specific patterns, rib lesion morphology, and clinical correlates. Data on uptake intensity, CT features, lesion number, location, tracer type, patient-specific risk factors, and follow-up behavior were extracted and analyzed. Results: Most solitary rib lesions are benign, particularly in low-risk patients or when located in the anterior/lateral arcs. Metastatic lesions are more likely to present as multiple foci, show cortical destruction on CT, exhibit high uptake intensity, and occur in patients with elevated PSA, high Gleason score, or ongoing androgen deprivation. ^18^F-PSMA-1007 is especially prone to UBU in the ribs compared to ^68^Ga-PSMA-11. Based on these variables, we propose a clinical decision tree to guide interpretation of PSMA-avid rib lesions. Conclusions: Accurate interpretation of rib lesions on PSMA PET/CT requires a multimodal, context-sensitive approach. Our diagnostic decision tree guides precise differentiation of benign versus metastatic rib lesions, enhancing staging accuracy and clinical decision-making. Biomarker-guided therapies offer potential for personalized treatment, though rib-specific validation remains a critical need.

## 1. Introduction

Prostate cancer (PCa) is the most frequently diagnosed malignancy in men and remains the second leading cause of cancer-related mortality worldwide. Advances in imaging techniques lead to detection of metastatic lesions in earlier stages, but interpreting these findings is challenging due to limited—yet rapidly accumulating—evidence on these modalities, while most existing studies rely on computed tomography (CT) scans and bone scintigraphy [1,2]. Approximately 70–90% of PCa metastases involve the skeletal system, predominantly affecting the pelvis and axial skeleton. These lesions are typically osteoblastic and disseminate hematogenously to red marrow-rich sites, fostering a favorable environment for tumor growth [3,4,5].

The advent of prostate-specific membrane antigen (PSMA) positron emission tomography/computed tomography (PET/CT) has revolutionized PCa imaging. Compared to conventional modalities such as bone scintigraphy and contrast-enhanced CT, PSMA PET/CT demonstrates significantly greater sensitivity and specificity. It has proven particularly valuable for staging high-risk PCa and detecting early recurrence sites following definitive therapy [6,7]. Nevertheless, like all imaging techniques, PSMA PET/CT is not without limitations, especially due to the phenomenon of unspecific bone uptake (UBU) [8].

UBU is characterized by focal PSMA tracer accumulation in bone in the absence of definitive radiographic or clinical evidence of metastasis. Rib lesions are a particularly common and challenging manifestation of UBU in PCa imaging. Although PSMA-targeted radiotracers, such as ^18^F-PSMA-1007, offer enhanced sensitivity for detecting metastatic disease, their specificity can be compromised by benign PSMA-avid lesions, resulting in false positives [9,10]. Reports indicate that UBU occurs in 30–57% of PSMA PET/CT scans, yet histopathologic confirmation reveals that only 9.1% of biopsied UBUs are truly metastatic [11,12]. These findings highlight the potential of UBUs to generate false-positive results, potentially leading to over-staging and misinformed therapeutic decisions [7,10,13,14].

A systematic review by Rizzo et al. revealed that ribs are involved in approximately 40% of UBU cases on PSMA PET/CT, and most isolated rib uptakes are non-metastatic in nature [8]. Accurately distinguishing between benign and malignant rib involvement is clinically crucial: a diagnosis of metastatic rib lesions can shift staging from oligometastatic to polymetastatic disease or from low-volume to high-volume PCa, necessitating a fundamental change in therapeutic approach. Furthermore, such misinterpretation may lead to erroneous diagnoses of recurrence following curative treatment [15,16,17].

This comprehensive review synthesizes the existing literature on the pathophysiology, diagnostic challenges, and imaging interpretation of PSMA-avid rib lesions in PCa, with a focus on distinguishing benign from malignant uptakes to enhance staging accuracy and promote individualized patient care. Unlike prior reviews, this manuscript provides a unique synthesis of evidence across diverse PCa states, including biochemical recurrence, non-metastatic castration-resistant prostate cancer, and oligometastatic disease. We propose a novel diagnostic decision tree that integrates lesion characteristics, clinical context, and tracer-specific patterns to guide precise differentiation of benign versus metastatic rib lesions, addressing a critical gap in standardized interpretive frameworks. By emphasizing therapeutic translation, this review highlights how accurate interpretation can optimize metastasis-directed therapies, prevent overtreatment, and provide a practical approach for clinicians navigating the complexities of PSMA PET/CT imaging [8,18].

## 2. Materials and Methods

To ensure a comprehensive and relevant evidence base for this review, studies were systematically selected based on a structured search strategy and predefined inclusion criteria. A literature search was conducted in PubMed, Embase, and Google Scholar, covering publications up to July 2025, using following keywords: (“prostate cancer” OR “prostate carcinoma” OR “prostatic neoplasms”) AND ((“rib” OR “costal” OR “costovertebral”) AND (“metastasis” OR “bone metastasis” OR “skeletal me-tastasis”)) AND (“PSMA PET” OR “PSMA PET/CT” OR “68Ga-PSMA-11” OR “18F-PSMA-1007” OR “18F-DCFPyL” OR “18F-rhPSMA7.3” OR “99mTc-PSMA” OR “Posi-tron Emission Tomography Computed Tomography” OR “Single Photon Emission Com-puted Tomography”) AND (“diagnosis” OR “detection” OR “sensitivity” OR “specificity” OR “false positive” OR “false negative” OR “accuracy”). Additional studies were identified through reference lists and clinical trial registries. Inclusion criteria comprised: (1) studies reporting on PSMA-avid rib lesions or bone metastases in PCa, with specific mention of ribs where possible; (2) clinical studies with diagnostic or therapeutic outcomes, including sensitivity, specificity, survival, or skeletal-related event data; (3) studies published in English with peer-reviewed data; and (4) case reports were included only if they contributed substantial or novel clinical insights into PSMA-avid rib lesions. Exclusion criteria included studies lacking rib-specific or PSMA-related data and non-peer-reviewed sources. Study quality was assessed based on sample size, methodology, and clinical relevance. From an initial pool of 1234 articles, 82 studies were selected, prioritizing those with rib-specific findings or high-impact outcomes. This approach ensured a robust evidence base focused on PSMA-avid rib lesions, addressing diagnostic, therapeutic, and pathophysiological aspects while acknowledging the scarcity of rib-specific data.

## 3. Biology and Clinical Translation of PSMA-Avid Rib Lesions in Prostate Cancer

### 3.1. Etiology and Pathophysiology of PSMA-Avid Rib Lesions

The predilection of PCa to metastasize to bone stems from a complex interplay between tumor-intrinsic properties and the specialized microenvironment of the skeletal system. This process begins with epithelial–mesenchymal transition (EMT), during which tumor cells downregulate E-cadherin and upregulate N-cadherin and vimentin, acquiring enhanced migratory and invasive capabilities [19].

The “seed and soil” hypothesis provides a conceptual framework for this organotropism: bone serves as a fertile “pre-metastatic niche”, enriched with chemokines (like CXCL12 and TGF-β) and adhesion molecules (e.g., integrins, cadherins) that facilitate the homing and anchoring of circulating tumor cells [20].

Once established within the bone, PCa cells exploit the “vicious cycle” of bone remodeling. They secrete parathyroid hormone-related protein, interleukin-6, and Wnt pathway inhibitors such as DKK1 and sclerostin, promoting osteoclast differentiation through receptor activator of nuclear factor kappa-Β ligand signaling. Osteoclast-mediated bone resorption subsequently releases TGF-β, IGF-1, and calcium, which further stimulate tumor proliferation. Concurrently, PCa cells induce osteoblast activation by secreting endothelin-1, bone morphogenetic proteins, and fibroblast growth factors. This dual activation of bone resorption and formation leads to the formation of characteristic osteosclerotic (osteoblastic) lesions [21,22,23].

This multifactorial mechanism explains why up to 90% of patients with advanced PCa develop bone metastases, with a predilection for the spine (68%), pelvis (42%), and ribs (15–20%) [24].

### 3.2. Therapeutic Targeting and Biomarkers for PSMA-Avid Rib Lesions

Several molecular pathways have shown promise in preclinical studies for drug targeting. The RANKL/RANK pathway, critical for osteoclast-mediated bone resorption, is a prime target, with denosumab reducing tumor burden in mouse models of osteoblastic metastases [21,22,23]. However, its efficacy in rib-specific lesions requires further clinical validation, and long-term use carries risks of osteonecrosis. The Wnt pathway, modulated by inhibitors like DKK1 and sclerostin, promotes bone formation and has shown preclinical efficacy in stabilizing osteoblastic rib lesions, though off-target effects limit translatability [3,25]. PSMA-targeted radioligand therapies, such as ^177^Lu-PSMA-617, demonstrate strong preclinical efficacy in targeting PSMA-avid rib lesions, but heterogeneous PSMA expression in CRPC may reduce effectiveness [26,27]. TGF-β signaling, a key mediator of tumor–bone interactions, is another promising target, with inhibitors like galunisertib showing reduced metastasis progression in preclinical models, though clinical trials are needed to confirm rib-specific efficacy [28,29].

Translation from preclinical models to clinic has been uneven for pathways driving PCa rib metastases. For RANKL/RANK, denosumab reduces skeletal-related events in mCRPC bone metastases but failed OS endpoints in prevention trials [21,30]. ^177^Lu-PSMA-617 succeeded in VISION and PSMAfore in mCRPC patients [31,32]. TGF-β inhibitors like galunisertib also show promise in combinations [33,34]. These efforts underscore the potential for rib lesion control but reveal gaps in site-specific efficacy data.

Biomarkers are critical for tailoring therapies to PSMA-avid rib lesions. PSMA expression (SUVmean >10 on PET/CT) predicts response to ^177^Lu-PSMA-617, with high uptake guiding MDTs like SBRT or radioligand therapy, though heterogeneous expression and tracer variability pose challenges [31,35]. Bone turnover markers reflect osteoblast/osteoclast activity, identifying candidates for denosumab, but lack rib specificity. Circulating tumor cell (CTC) counts (>5 cells/7.5 mL) and ctDNA (such as AR-V7) predict ARPI resistance, favoring PSMA-targeted therapies for rib lesions, yet require standardized assays [36,37]. TGF-β1 (>200 pg/mL) and p-SMAD2/3 signal response to TGF-β inhibitors, but non-specificity and biopsy needs limit use [38]. Rib-specific biomarker data and standardized thresholds remain critical gaps, necessitating prospective validation.

While mechanistic studies provide valuable insights into the molecular drivers of bone metastasis, much of the evidence is derived from in vitro and in vivo models, which may not fully capture the complexity of human disease. Animal models often fail to replicate the heterogeneous tumor microenvironment of prostate cancer, and rib-specific metastatic mechanisms remain underexplored due to limited histopathological data. Furthermore, most preclinical studies focus on axial skeletal metastases, leaving rib-specific mechanisms underexplored. The heterogeneity of PCa and limited clinical validation of these pathways highlight the need for prospective trials to translate these findings into effective therapies for rib metastases. These gaps highlight the need for clinical studies to validate preclinical findings and elucidate rib-specific metastatic pathways.

## 4. Rib Involvement in Prostate Cancer

Metastatic involvement of the ribs in prostate cancer typically occurs as part of widespread skeletal disease. Compared to vertebral or pelvic metastases, rib lesions are more often asymptomatic in early stages, though they may eventually present with localized pain, palpable masses, or pathological fractures [39,40]. Autopsy and PSMA PET/CT studies confirm that rib involvement typically signals multifocal disease, with 97.9% of metastatic rib lesions co-occurring with nodal or other bone metastases, guiding systemic therapy over localized approaches. These correlations highlight the importance of comprehensive imaging to assess disease extent and inform treatment planning [17,39,41]. These lesions are commonly detected using computed tomography (CT), magnetic resonance imaging (MRI), or PSMA PET/CT, which provide superior spatial resolution compared to traditional bone scintigraphy [42].

Solitary rib metastases are uncommon in PCa, particularly in the context of oligometastatic disease [43]. When a solitary PSMA-avid rib lesion is identified, it is critical to exclude benign etiologies before concluding metastatic spread [18]. Although rare, solitary rib metastasis should prompt careful investigation to rule out alternative diagnoses or to confirm systemic dissemination [44,45]. Numerous benign and malignant conditions have been associated with rib lesions, and a comprehensive list of these differential diagnoses along with their radiological characteristics is provided in Table 1. Further study-specific details categorized by diagnosis and pattern of involvement are presented in Supplementary Tables S1–S5.

According to the original CHAARTED trial definition, the ribs are classified as extra-axial sites, and their involvement may suggest high-volume metastatic disease [15]. However, subsequent evidence indicates that the specific site of metastasis is not an independent prognostic factor in patients with oligometastatic PCa [112]. More recently, emerging data propose that solitary rib metastases might represent a unique oligometastatic phenotype with a favorable prognosis, especially when managed aggressively. Median overall survival (OS) in such cases ranges from 45 to 60 months when treated with metastasis-directed therapy (MDT) such as stereotactic body radiotherapy (SBRT) or surgery combined with systemic treatment [15,113].

Nevertheless, due to the absence of prospective trials focusing specifically on rib metastases in PCa, individualized management strategies based on multidisciplinary team assessment remain essential.

## 5. Diagnostic Modalities of Rib Involvement in Prostate Cancer

### 5.1. CT Scan: Structural Assessment with Limitations

**Figure 1:**
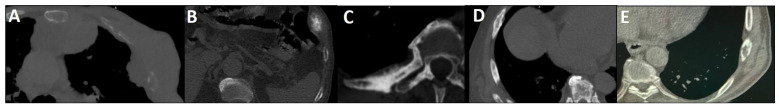
Spectrum of CT changes in prostate cancer patients, arranged by highest risk of malignancy (**A**–**E**). Expansile lytic-destructive lesion with a “soft tissue component”, indicating aggressive variant prostate cancer or concurrent malignancy (**A**). Sclerotic-destructive metastases with “cortical expansion” (**B**). Sclerotic metastases showing sun-burst “periosteal reaction” and pathological fracture (**C**). Sclerotic metastases with “cortical thickening and irregularity” (**D**). Lytic–sclerotic metastasis featuring “focal small areas of cortical erosion/destruction” (**E**).

**Figure 2:**
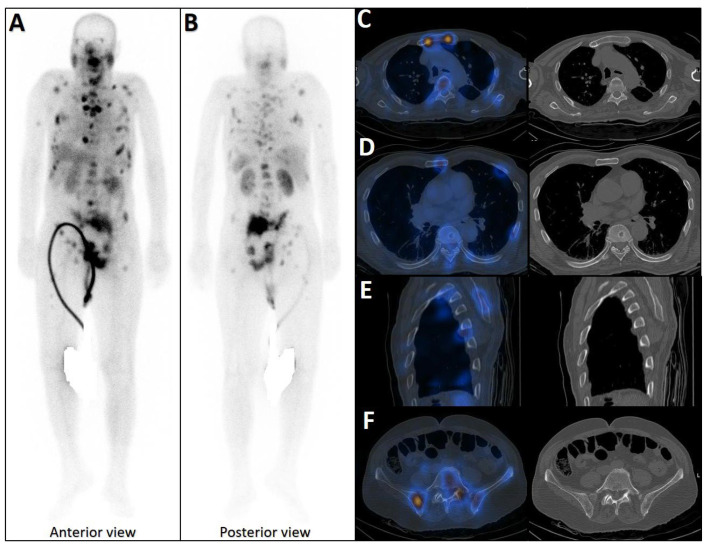
Early phase of metastases without clear sclerotic changes in the bone. (**A**) 70-year-old man with elevated PSA was recently diagnosed with prostate adenocarcinoma (all cores positive with Gleason score 5 + 4) and referred for staging. The whole-body 99mTc-PSMA scan (**A**,**B**) and SPECT/CT showed multiple PSMA-avid skeletal metastases, most of which showing no/minimal density changes (**C**–**F**).

**Figure 3:**
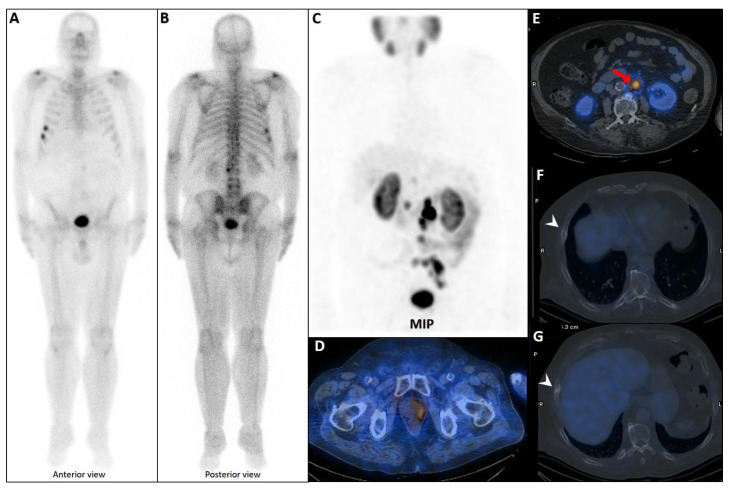
False positive findings in bone scintigraphy due to rib fractures. A 78-year-old man with prostate adenocarcinoma (biopsy Gleason score 4 + 4, serum PSA = 56.8 ng/mL) was referred for staging. Whole-body bone scan revealed two consecutive focal osteoblastic activities in the lateral arc of the ribs on the right side (**A**,**B**). 99mTc-PSMA SPECT/CT (**C**) revealed increased tracer uptake in the prostate gland (**D**), multiple metastatic lymphadenopahies on the left side of pelvis and para-aortic regions (red arrow, (**E**)). Moreover, fractures in the right 6th and 7th ribs showed no PSMA avidity (white arrowheads, (**F**,**G**)).

CT is widely utilized for evaluating rib metastases due to its high spatial resolution and capacity to detect cortical destruction, sclerosis, and soft tissue extension (Figure 1). It is particularly effective in identifying osteoblastic lesions, which are common in PCa, and serves as a valuable tool for guiding biopsies. However, CT has notable limitations: it exhibits low sensitivity for detecting early metastatic lesions (those lacking structural changes (Figure 2) and offers limited specificity in differentiating benign findings (e.g., post-traumatic sclerosis) from malignant ones (Figure 3) [8,114,115].

### 5.2. Bone Scintigraphy: High Sensitivity but Lack of Specificity

Bone scintigraphy, typically using 99mTc-methylene diphosphonate (MDP), is becoming an outdated or even obsolete method for detecting skeletal metastases, including rib involvement [100,116,117]. Historically, its strengths included high sensitivity, as it could identify osteoblastic activity before structural alterations become evident, and the ability to screen the entire skeleton in a single session. However, its specificity was limited, and it frequently yielded false positives, as degenerative changes, healing fractures (Figure 3), or infections can mimic metastatic lesions [118]. Furthermore, it cannot reliably differentiate PCa metastases from other malignant or benign bone conditions [119].

### 5.3. Multiparametric MRI: High Sensitivity with Diagnostic Challenges

Multiparametric MRI (mpMRI), incorporating T1-weighted, T2-weighted, diffusion-weighted imaging (DWI), and dynamic contrast-enhanced sequences, demonstrates high sensitivity (approximately 84%) for detecting rib metastases in PCa, particularly at early stages involving bone marrow infiltration before overt structural changes emerge [120,121,122]. It is especially effective in distinguishing malignant lesions (characterized by low T2 signal and restricted diffusion) from benign entities such as fractures or hemangiomas, which typically exhibit high T2 signal. Additionally, mpMRI offers excellent soft-tissue resolution and characterization [121,123].

Despite its diagnostic strengths, mpMRI is not without limitations. It is associated with high costs, restricted availability, and a propensity for false positives, especially in the context of post-treatment changes or inflammatory processes that can mimic malignancy. Moreover, patient-specific factors such as claustrophobia, metallic implants, or poor tolerance of long scan durations can hinder its widespread application [124,125].

### 5.4. Ultrasonography: Complementary Characterization of Rib Metastases

Ultrasonography (US) is rarely used for routine diagnosis of rib metastases in prostate cancer due to lower sensitivity than PSMA PET/CT, which better detects bone metastases. However, high-resolution US can complement bone scintigraphy by characterizing rib lesions. A prospective study by Lee et al. showed that osteoblastic prostate cancer rib metastases have mild cortical irregularity without soft-tissue mass or bone destruction, unlike osteolytic metastases from renal cell carcinoma that cause extensive cortical damage. US is valuable in high-risk prostate cancer patients to confirm solitary rib metastases, potentially avoiding biopsies. It is also useful when CT results are inconclusive or radiation exposure should be minimized. Further research is needed to standardize its use [126,127].

### 5.5. PSMA PET/CT: Superior Accuracy but Not Perfect

**Figure 4:**
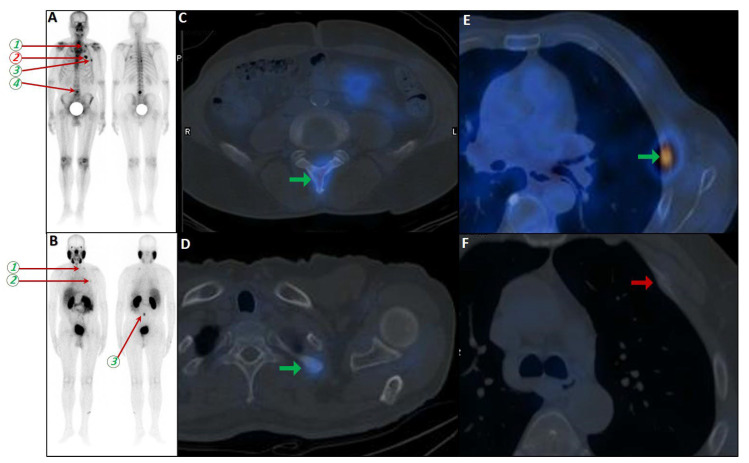
Partial concordance of PSMA and bone scan. A 66-year-old man with prostate adenocarcinoma (biopsy Gleason score 4 + 5 in 8/12 cores, serum PSA = 45 ng/dL) was referred for staging. Bone scan showed four foci of increased osteoblastic activity, three in the ribs (non-consecutive, anterior/lateral aspect) on the left side as well as L5 vertebra (**A**). Whole-body 99mTc-PSMA scan SPECT/CT (**B**) showed PSMA-avid lymph node metastases in the left external iliac and left common iliac (not shown). Skeletal metastases (green arrow) in the L5 spinous process (**C**) and posterior aspect of the left 2nd rib (**D**) and lateral 5th left rib (**E**), and non-PSMA-avid fracture in the 4th left rib (red arrow, (**F**)). The patient was down-staged from polymetastatic high-volume disease to oligometastatic disease.

PSMA imaging has transformed the staging landscape of PCa due to its high specificity for PSMA-expressing tumor cells and superior diagnostic accuracy relative to conventional modalities like CT and bone scintigraphy (Figure 4) [128]. By combining molecular imaging (PET) with anatomic localization (CT), this modality facilitates precise lesion characterization and localization. It is particularly valuable in detecting oligometastatic disease and in guiding metastasis-directed therapy [129]. This makes it ideal for guiding metastasis-directed therapies like surgery or radiotherapy and assessing target expression for 177Lu-PSMA-617 treatments [130].

Nevertheless, PSMA PET/CT is not immune to false positives. Uptake can occur in benign bone conditions (e.g., Paget’s disease, healing fractures, vertebral hemangiomas), non-prostatic PSMA-expressing tissues (e.g., sympathetic ganglia), and non-PCa malignancies (e.g., colorectal or hepatocellular carcinomas), all of which can complicate image interpretation [18]. These pitfalls underscore the need for correlating imaging findings with clinical context and additional modalities when necessary.

## 6. Pitfalls in UBU Diagnosis

### 6.1. High Incidence of UBUs and False Positives

PSMA PET/CT frequently detects UBUs, particularly in the ribs, where they are often misinterpreted as metastases, especially by less experienced readers [40]. Various PSMA tracers including ^18^F-PSMA-1007, ^68^Ga-PSMA-11, and ^18^F-Piflufolastat exhibit differing propensities for UBU detection.

As reported by Rizzo et al., UBUs occur predominantly in the ribs, with ^18^F-PSMA-1007 showing the highest overall incidence (11.6% to 71.7%), with 55% of those foci located in the ribs. In comparison, ^68^Ga-PSMA-11 demonstrates a lower overall UBU incidence (0% to 23.9%), 54% of which involve the ribs. Similarly, ^18^F-DCFPyL presents a total UBU incidence of 19.8%, with 44% of cases affecting the ribs [8].

These PSMA-avid osseous lesions often reflect benign processes rather than true metastatic disease. Common etiologies include fibroblastic repair reactions, fibrous dysplasia, healing fractures, Paget’s disease, and vertebral or rib hemangiomas. Grünig et al. also suggested that isolated bone marrow islands, particularly in the ribs and extremities, may contribute to UBU, potentially due to activated granulocytes in the bone marrow. These islands can be mistaken for pathological lesions on imaging, such as metastases, due to their focal uptake of radiotracers like ^18^F-PSMA-1007 in PET scans, but they are typically benign and may result from physiological processes like marrow reconversion or inflammation [12]. For example, Ou et al. reported a positive predictive value (PPV) of only 26% for rib biopsies performed on ^18^F-Piflufolastat-avid foci, with 52% of biopsy-negative lesions located in the ribs [131]. Similarly, Orevi et al. found that among post-prostatectomy patients with undetectable PSA levels, 41.2% had false-positive PSMA-avid rib foci (with SUVmax < 7), consistent with non-specific uptake [93].

Further corroborating this, Vollnberg et al. found that only 9.1% of biopsied UBU rib foci using ^18^F-PSMA-1007 were confirmed as metastatic, and the benign lesions showed no PSMA expression histologically [11]. These findings underscore the high false-positive rate associated with rib UBUs, which can lead to overdiagnosis, misclassification, and unnecessary systemic treatment.

Table 2 provides a comparative summary of UBU incidence rates, metastasis confirmation rates, and tracer-specific diagnostic performance, alongside their radiologic features and differential diagnostic considerations.

### 6.2. Equivocal Lesions and Lack of CT Correlates

Equivocal bone lesions, often categorized as PSMA-RADS-3B, present a significant diagnostic challenge, especially when localized to the ribs, where benign and malignant etiologies frequently overlap. A study by Mainta et al. reported that 55.8% of bone lesions on ^68^Ga-PSMA-11 PET/CT were classified as equivocal, with 48.8% of these involving ribs, and many lacking corresponding CT abnormalities, thereby necessitating follow-up imaging or biopsy for clarification [99].

The study by Simsek et al. conducted a retrospective head-to-head comparison of bone scintigraphy, bone scintigraphy with SPECT/CT, and ^68^Ga-PSMA-11 PET/CT in 138 PCa patients to evaluate their efficacy in detecting bone metastases. The study notes that the CT component aided ^68^Ga-PSMA-11 PET/CT in morphological correlation, contributing to its superior specificity and accuracy by providing anatomic and morphologic information. The result implies that CT correlation is crucial in both modalities but is particularly effective in ^68^Ga-PSMA-11 PET/CT [100].

A systematic review by Woo et al., encompassing 22 studies, found a 20% pooled prevalence of equivocal bone lesions using PSMA PET/CT, but only 28% were ultimately confirmed as metastatic, emphasizing the difficulty of distinguishing benign from malignant uptake in such cases. This ambiguity is particularly pronounced in rib lesions, where histopathologic studies report a metastasis rate as low as 2% for UBUs, raising concerns over whether isolated PSMA-avid rib lesions should even be routinely categorized as PSMA-RADS-3B [8,45].

### 6.3. False Negatives Due to Heterogeneous PSMA Expression

**Figure 5:**
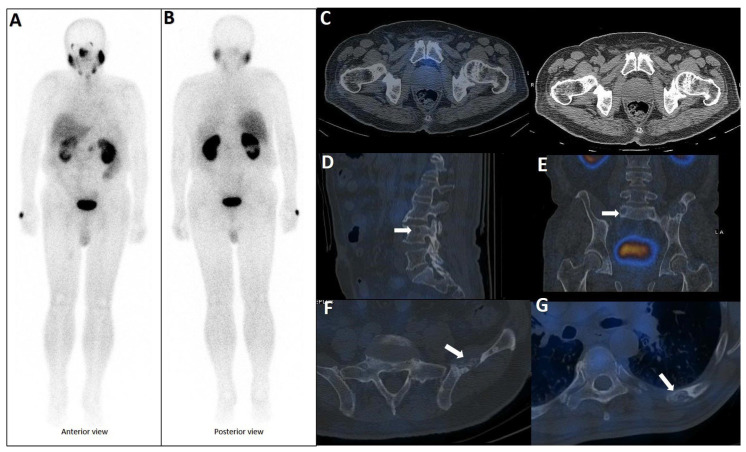
PSMA scan ruling out the possibility of metastatic prostate cancer. A 73-year-old man was referred for diagnosis/staging, due to mildly elevated serum PSA levels, and a few lytic–sclerotic bone lesions incidentally found in the recent abdominopelvic CT scan. Whole-body 99mTc-PSMA (**A**,**B**) and SPECT/CT (**C**) showed faint PSMA uptake in the prostate gland (PRIMARY score 1) with multiple non-PSMA-avid (white arrows) lytic–sclerotic changes in the L4 vertebra (**D**), sacrum (**E**), left iliac (**F**) and left 6th rib (**G**). No nodal or visceral involvement was evident. These bone lesions were finally concluded as unrelated pathology to prostate cancer, yet the rare possibility of non-PSMA-avid tumor could not be entirely ruled out.

False-negative findings in PSMA PET/CT can occur due to heterogeneous PSMA expression and partial volume effects, particularly in castration-resistant prostate cancer (CRPC) patients with small-volume rib metastases [145,146] and rarely in the staging scenario [147] (Figure 5). As highlighted by Serani et al., diminished PSMA expression in CRPC may obscure metastatic lesions, reducing detection sensitivity. In support, Simsek et al. reported that 1.4% of ^68^Ga-PSMA-11 scans failed to identify existing metastases due to this limitation [100,132].

Additionally, treatment-induced modulation of PSMA expression, as noted by both Simsek et al. and Rizzo et al., complicates post-therapy imaging interpretation, especially in patients undergoing hormonal or systemic therapies. These false negatives carry the risk of underdiagnosis, which may result in delayed initiation of critical interventions in patients with true metastatic progression [8,100,148].

### 6.4. Conflicting Results Among Different Studies

Conflicting results across studies complicate the interpretation of PSMA-avid rib lesions, such as Woo et al., who report a 20% prevalence of equivocal lesions with only 28% confirmed as metastatic, while Fragkiadaki et al. suggest a higher malignancy rate [17,140]. These discrepancies may stem from differences in tracer types, patient risk profiles, or imaging protocols. Moreover, many studies rely on retrospective designs or imaging-based follow-up, which may introduce bias compared to biopsy-confirmed diagnoses. The lack of standardized SUV thresholds further contributes to diagnostic variability, representing a critical gap in the field [11,18].

## 7. Approaching Rib Involvement in Prostate Cancer

### 7.1. Clinical Perspective

PSMA-avid rib lesions play a critical role across various disease states, including biochemical recurrence (BCR), non-metastatic castration-resistant prostate cancer (nmCRPC), and oligometastatic disease, influencing diagnostic accuracy and therapeutic decisions. In the setting of BCR, characterized by rising PSA levels after definitive therapy without overt radiographic disease on conventional imaging, PSMA-avid rib lesions are frequently false positives, particularly when solitary, with 60–80% being benign. Clinicians often proceed with salvage radiotherapy to the prostate bed, with or without pelvic lymph node irradiation, combined with androgen deprivation therapy (ADT), typically disregarding solitary lesions [149]. However, when metastatic rib involvement is confirmed through serial PSA monitoring and PSMA PET/CT, rather than conventional CT, MDT such as SBRT may be justified to delay systemic treatment, preventing both undertreatment and overtreatment [113,150,151].

In nmCRPC, PSMA PET/CT detects occult metastatic sites, including rib lesions, in 50–60% of patients [42,149]. While solitary rib lesions often remain benign, confirmed metastatic spread reclassifies the disease as metastatic castration-resistant prostate cancer (mCRPC), prompting the initiation of systemic therapies such as androgen receptor pathway inhibitors (ARPIs) [152,153,154], if not previously started in the BCR/nmCRPC state [154]. The potential role of MDT, such as SBRT to rib lesions, or pelvic irradiation in nmCRPC is still under investigation, highlighting the need for further studies to define optimal management strategies [155].

**Figure 6:**
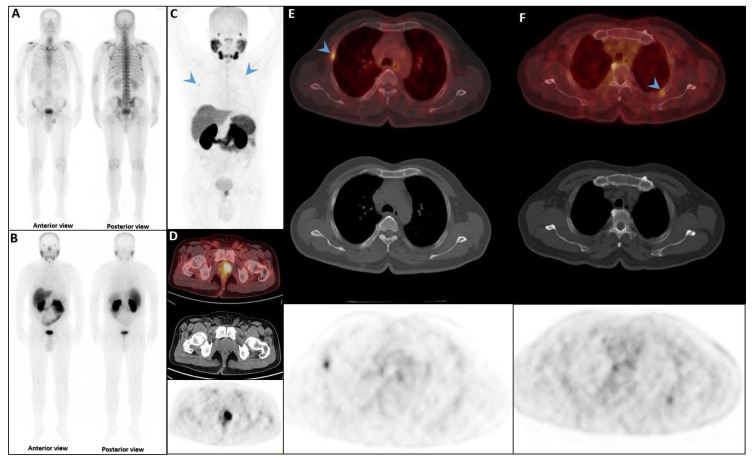
Complete discordant findings between PSMA and bone scan. 61-year-old male referred for staging that transrectal ultrasound-guided prostate biopsy showed GS 4 + 3 (12 of 12 cores involved), perineural invasion (+), and serum PSA level = 17 ng/mL. A whole-body bone scan revealed multiple increased tracer uptake in bilateral rib (**A**). 99mTc-PSMA SPECT/CT revealed no abnormal tracer uptake (**B**). Considering discordant findings in the 99mTc-PSMA and bone scan, the referring physician decided to request a 68Ga-PSMA PET/CT. The scan showed (**C**) multiple foci of increased activity in the left side of the prostate gland (**D**) as well as two foci of increased activity in bilateral ribs with no density changes on the corresponding CT images (SUVmax up to 3.84) (blue arrowhead, **E**,**F**). The rib lesions were finally considered as equivocal. Ultimately, the patient underwent RP and nadir PSA was 0.02 ng/mL.

In oligometastatic PCa, defined by a limited disease burden of up to five metastatic sites, the identification of rib lesions is pivotal for volume stratification and therapeutic planning. Only 10–20% of PSMA-avid rib lesions in this setting are truly metastatic [113]. A single metastatic rib lesion may classify the disease as low-volume, making it eligible for MDTs like SBRT to delay systemic treatment and disease progression while preserving quality of life [156]. Conversely, presence of at least three additional axial bone or concurrent visceral metastases may indicate high-volume disease, necessitating aggressive systemic therapies such as ADT, ARPIs, and possibly docetaxel [150,157]. The presence and pattern of rib lesions thus create a clinical dichotomy, underscoring the importance of tailored management guided by advanced imaging, such as PSMA PET/CT, and multidisciplinary decision-making (Figure 6) [158]. Accurate characterization of rib lesions across these settings is essential to balance the risks of overtreatment and undertreatment, ensuring optimal patient outcomes.

### 7.2. A Radiology Perspective

**Figure 7:**
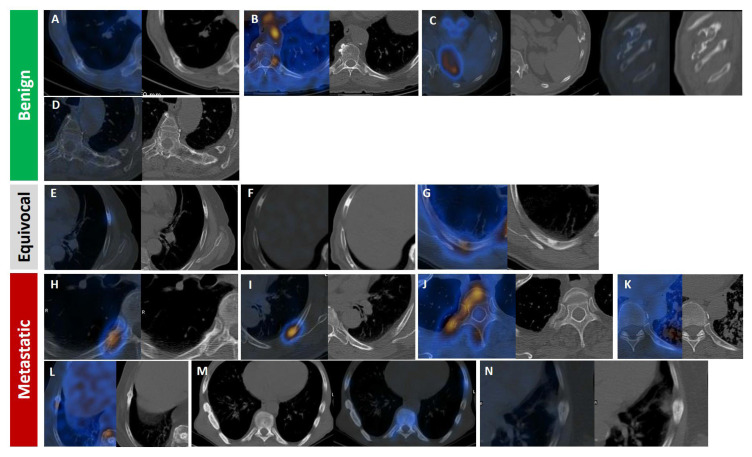
Radiologic appearance of PSMA-avid rib lesions in prostate cancer patients, categorized by likelihood of malignancy: benign (**A**–**D**), equivocal (**E**–**G**; all isolated), and malignant (**H**–**N**). Fracture line with callus formation and faint PSMA uptake (**A**). Focal moderate uptake at the costovertebral joint, consistent with degenerative changes (**B**). Well-defined expansile non-PSMA-avid lytic lesion at the costochondral junction, likely a benign chondroid lesion (**C**). Left transverse process connected to an adjacent isolated rib (**D**). Focal lytic–sclerotic lesion anterolaterally with moderate uptake. (**F**) Focal dense sclerosis without uptake (**E**). Eccentric/mural focal sclerosis with moderate uptake (**G**). Focal intense uptake with periosteal reaction and sclerosis at the costovertebral rib portion (**H**). Focal intense uptake in the posterior rib arc with ground-glass appearance (**I**). Focal intense uptake with mild sclerosis in the vertebral body extending to head, neck, and posterior rib arc (**J**). Focal mild uptake with sclerotic changes in posterior arc amid miliary lung metastasis (**K**). Linear sclerotic lesion showing moderate PSMA uptake and callus formation, indicating pathologic fracture (**L**). Linear dense sclerotic changes along lateral rib arc with mild PSMA uptake (**M**). Periosteal reaction with faint PSMA uptake and adjacent soft tissue involvement (**N**).

To enhance diagnostic precision, there is a growing consensus on the need to establish standardized evaluation frameworks and to integrate quantitative and qualitative imaging features with clinical context. Complete list of references containing figures with PSMA-avid rib lesion is presented in Supplementary Table S6. Based on a comprehensive review of the literature, several key indicators have emerged as useful tools in evaluating PSMA-avid rib lesions and understanding their clinical implications. These indicators are summarized in Table 3. Moreover, we have reviewed the radiologic appearance of rib lesions in our cohort of patients with prostate cancer (350 SPECT/CT and 80 CT scan) and categorized them into benign, equivocal or malignant appearance (Figure 7).

#### 7.2.1. Radiotracer Uptake Intensity

Quantitative metrics such as SUVmax and SUVpeak are commonly used in PSMA PET/CT interpretation; however, they often lack sufficient specificity for distinguishing metastatic from benign rib lesions.

Arnfield et al. proposed an SUVmax threshold of <7.2 to suggest benignity in lesions detected with ^18^F-PSMA-1007, yet lesions falling within the equivocal range (SUVmax 7.2–11.1) frequently overlapped with metastatic disease, thereby reducing diagnostic clarity [13]. Similarly, Chiu et al. reported low specificity values (31–77%) for SUV cutoffs in bone lesions, including ribs, using ^68^Ga-PSMA-11 PET/CT [92].

Further insights by Ou et al. revealed that SUVpeak values were significantly higher in biopsy-positive lesions (mean 17.3) compared to biopsy-negative ones (mean 5.4). They also suggested a SUVpeak-to-liver SUVmean ratio cutoff of 1.7, which achieved 61% sensitivity and 92% specificity for metastatic disease. However, rib lesions in PSMA-PET/CT frequently exhibit a higher likelihood of being benign, contributing to a lower positive predictive value and an increased risk of false positives, particularly in conditions such as fibro-osseous lesions or Paget’s disease, despite variable SUV values [131].

In line with these findings, Rizzo et al. observed that SUVmax values for UBUs typically range from 3.4 to 7.7, generally lower than those seen in confirmed metastases, but substantial overlap persists, complicating interpretation [8]. These data collectively underscore the limited reliability of SUV-based thresholds, especially when evaluating solitary or equivocal rib lesions, where clinical context and multimodal correlation remain essential.

In studies using ^18^F-PSMA-1007, SUVmax values below 7 to 7.2 are typically considered benign, while values between 7.2 and 11.1 fall into an equivocal range. Values exceeding 11.1 are more suggestive of malignancy. For ^68^Ga-PSMA-11, rib lesions with SUVmax below 5 or lesion-to-blood pool SUV ratios less than 2.2 are generally non-malignant. Again, these thresholds are not absolute, and interpretation must be informed by clinical context and findings from other imaging modalities. For example, in post-radical prostatectomy patients with undetectable PSA, false positives are more common due to benign PSMA uptake [92,131,135,143].

#### 7.2.2. Number of Lesions

The quantity and distribution of PSMA-avid rib lesions serve as critical indicators for assessing metastatic involvement in PCa [17,40,135,142]. Solitary rib lesions with PSMA uptake are predominantly benign, with approximately 90% of cases attributed to non-malignant causes such as fractures, post-radiation changes, or other benign conditions rather than true metastatic disease [11,40,97]. On the other hand, in confirmed oligometastatic settings, MDTs like SBRT may delay the need for systemic treatment, while benign lesions typically require only clinical observation [142,159]. Accurate CT correlation (Section 7.2.4) is crucial in these cases to avoid misdiagnosis [14,160]. The application of standardized interpretive frameworks, such as PSMA-RADS and PROMISE, or a combination of both, improves diagnostic specificity in oligometastatic cases and reduces the risk of misclassifying benign uptake as metastatic, ensuring more precise treatment planning and prognosis evaluation [7].

**Figure 8:**
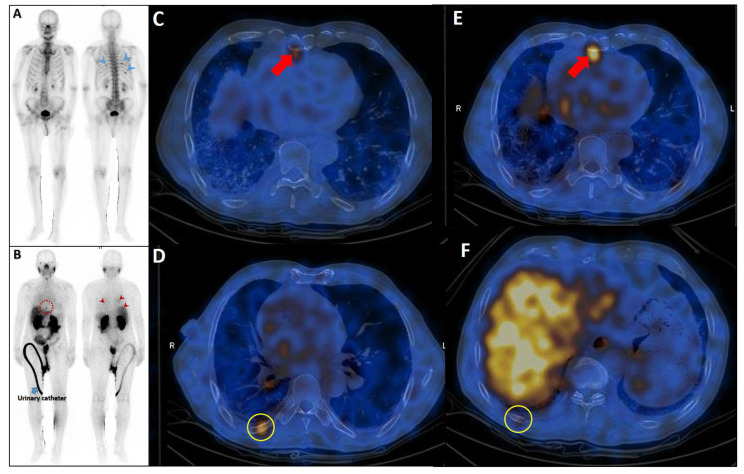
Complementary role of PSMA SPECT/CT to bone scan in a patient with equivocal rib metastases. A very-high-risk prostate cancer patient (Gleason score: 5 + 4, serum PSA = 43 ng/dL) was referred for staging. The bone scan revealed at least three foci of osteoblastic activity in the ribs posteriorly ((**A**), blue arrowheads). Subsequently, the patient underwent PSMA SPECT/CT (**B**–**F**), confirming a mild PSMA avidity in the mentioned regions ((**B**), red arrowheads) with subtle sclerotic changes in two of them ((**D**,**F**), yellow circles). Moreover, another focus of uptake was noticed in the xiphoid process ((**B**), dashed red circle; (**C**,**E**), red arrows). Given the patient’s clinical history, presence of lymph nodes metastases (not shown), and possibility of xiphoid involvement, the patient was finally concluded as having metastatic rib lesions in the bones.

In contrast, multiple rib lesions present a more complex diagnostic challenge, as they significantly increase the likelihood of metastatic disease, particularly in patients with advanced-stage PCa, elevated PSA levels, or high Gleason scores (Figure 8) [14,160]. Unlike solitary lesions, multifocal rib involvement raises greater suspicion for metastasis, especially when accompanied by lymph node involvement [17,40,135,142]. A polymetastatic distribution pattern further enhances diagnostic confidence among readers [99].

#### 7.2.3. Location

The anatomical site of rib lesions offers additional diagnostic insight, although PSMA-specific data remain limited. Lesions occurring in the lateral or anterior rib arcs are more often benign and frequently associated with trauma or degenerative processes, as previously observed in bone scans for breast cancer metastases [161]. In contrast, lesions located in the posterior ribs, particularly near the costovertebral junction, rib head, or neck, are more commonly linked to metastatic disease. This distribution pattern, noted in qualitative bone imaging of malignant tumors, suggests that lesion location may assist in risk stratification, though further studies using PSMA PET/CT are needed to quantify risk with precision [162].

#### 7.2.4. CT Morphology or Bone Scan

CT imaging morphology plays a pivotal role in characterizing PSMA-avid rib lesions. Lesions showing sclerosis with cortical destruction are highly indicative of metastasis, with an odds ratio (OR) of 22.3, whereas lesions that lack CT abnormalities are benign in approximately 80% of cases and often reflect healed fractures [17]. Additionally, lesions with well-defined sclerotic borders tend to be benign, and benign-appearing CT findings confer a 100% negative predictive value for malignancy. However, the positive predictive value of malignant features on CT remains lower at 44.4% [137]. In contrast, bone scintigraphy adds only marginal value (approximately 4%) in lesion characterization, reaffirming the central role of CT in diagnostic evaluation [7,100].

#### 7.2.5. Patient-Specific Factors

Patient-related clinical parameters significantly influence the risk assessment of PSMA-avid rib lesions. Factors such as advanced age, elevated PSA levels, Gleason grades 4–5 (OR = 4.15), and ongoing ADT at the time of PSMA PET (OR = 4.06) are all associated with increased probability of metastatic rib involvement [17,131,137]. Furthermore, shorter PSA doubling times and advanced tumor stages are predictive of metastatic spread, and these clinical characteristics should guide interpretation when evaluating rib uptake on PSMA PET/CT [163].

#### 7.2.6. Changes over Time

Longitudinal imaging follow-up of PSMA-avid rib lesions is a valuable strategy for differentiating benign from malignant findings. Lesions that remain stable in number, uptake intensity, and molecular tumor volume over time are usually benign. Studies show that 23–79% of UBUs remain unchanged on follow-up imaging, while 14–23% of cases progress and are later confirmed as metastatic [12,42,143]. Notably, low post-treatment PSA levels (defined as <0.1 ng/mL following prostatectomy or <2 ng/mL above the nadir after radiotherapy) strongly favor benignity and enhance diagnostic confidence [136,144] (Figure 6).

#### 7.2.7. Tracer Type

PSMA-targeted tracers differ in their biodistribution, pharmacokinetics, and propensity for non-specific rib uptake, influencing diagnostic performance and interpretive confidence.

^18^F-PSMA-1007, while offering high sensitivity and favorable imaging properties, exhibits biliary excretion and demonstrates a higher overall rate of non-specific rib uptake (36%) compared to ^68^Ga-PSMA-11, which shows a lower incidence (8%), but the malignancy confirmation rate is paradoxically lower for ^18^F-PSMA-1007 (8%) than for ^68^Ga-PSMA-11 (29%) [138]. This discrepancy, confirmed in intraindividual comparative studies, may be further exaggerated by digital PET systems, emphasizing the need for tracer-specific diagnostic thresholds [8].

### 7.3. Diagnostic Decision Tree for PSMA-Avid Rib Lesions

**Figure 9:**
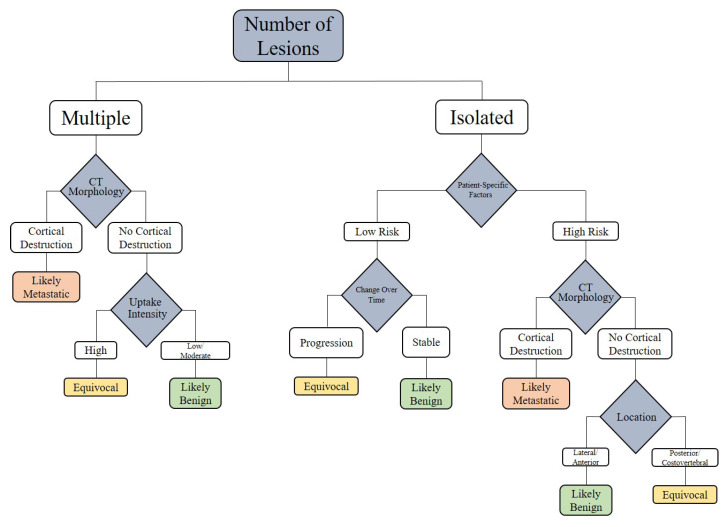
Approaching the PSMA-Avid Rib Lesion.

To translate the diagnostic parameters into a practical clinical workflow, we propose the following decision tree (Figure 9). This tool is based on lesion count, CT morphology, uptake intensity, lesion location, patient-specific factors, tracer type, and follow-up evolution. It provides a systematic guide to differentiate between benign and metastatic rib lesions detected on PSMA PET/CT.

The proposed diagnostic decision tree integrates evidence from multiple studies to guide treatment decisions for PSMA-avid rib lesions, synthesizing diagnostic parameters and clinical factors to differentiate benign from metastatic lesions. However, the scarcity of rib-specific data underscores the need for future case-based studies to validate the decision tree’s efficacy in diverse clinical scenarios, such as biochemical recurrence or mCRPC, to refine its impact on treatment outcomes.

While our proposed diagnostic decision tree integrates multiple parameters to enhance diagnostic precision, it is based primarily on retrospective studies with variable follow-up durations. The scarcity of prospective trials focusing specifically on rib metastases, as opposed to axial skeletal lesions, limits the generalizability of our framework. Future studies are needed to validate this approach and address the understudied role of rib-specific metastases in prostate cancer staging and management.

## 8. Conclusions

Differentiating PSMA-avid rib lesions as benign or metastatic remains a nuanced diagnostic challenge in prostate cancer imaging, particularly in the era of highly sensitive PSMA PET/CT. While the ribs are frequently involved in UBU, the vast majority of solitary rib lesions are benign, often attributable to trauma, degenerative change, or non-malignant marrow processes rather than true metastatic spread.

Our review synthesizes emerging evidence and proposes a structured diagnostic framework that incorporates multiple parameters: lesion count, tracer uptake intensity, CT morphology, anatomical location, clinical risk factors, temporal progression, and tracer type. Among these, CT morphology and number of lesions emerge as the most reliable discriminators, with cortical destruction and multifocal involvement strongly favoring metastatic disease. Conversely, isolated lesions with low to moderate uptake, benign CT features, anterior/lateral rib location, low-risk clinical profiles, and stability on follow-up imaging are highly suggestive of benignity.

Tracer-specific characteristics also influence interpretive confidence, as ^18^F-PSMA-1007 is more prone to false positives than ^68^Ga-PSMA-11 or ^18^F-DCFPyL. Therefore, interpretation must be tailored not only to imaging features but also to patient context and tracer bio-distribution.

This review advances the field by offering a comprehensive analysis of PSMA-avid rib lesions. Our proposed diagnostic decision tree represents a novel tool for standardizing interpretation, reducing diagnostic ambiguity, and guiding personalized treatment strategies. By bridging diagnostic and therapeutic perspectives, this work provides a roadmap for improving staging accuracy and optimizing outcomes in prostate cancer patients with suspected rib involvement.

Recognizing the diagnostic ambiguity of PSMA-avid rib lesions, particularly in oligometastatic and BCR states, we propose an integrative decision tree to aid clinicians and radiologists in stratifying these findings. This approach may prevent unnecessary biopsies, avoid overtreatment, and support appropriate MDT when warranted.

Advancing the management of PSMA-avid rib lesions in PCa requires addressing critical research gaps through innovative approaches. Multi-omics profiling, integrating genomics, proteomics, and metabolomics, could elucidate rib-specific molecular drivers and biomarkers, refining diagnostic algorithms and patient stratification for therapies. Spatial transcriptomics offers potential to map gene expression within the rib lesion microenvironment, revealing metastatic niches and validating PSMA PET/CT findings, though tissue access and technical complexity pose challenges. High-priority research directions include rib-focused clinical trials, prospective validation of biomarkers, and standardized imaging molecular correlations to enhance precision diagnostics and personalized treatment for PSMA-avid rib lesions.

## Figures and Tables

**Table 1:** Differential Diagnoses and Imaging Characteristics of PSMA-avid rib lesions.

Diagnosis *	Radiologic Appearance	References (Intense Uptake) **
Prostate Carcinoma Metastasis (including Bone Metastases)	Lytic or sclerotic lesion, often multiple; may show osteolytic, osteoblastic, or mixed patterns; soft-tissue mass in some cases; aggressive appearance with cortical disruption	[7,12,13,26,40,46,47,48,49,50,51,52,53,54,55,56,57,58,59,60,61,62,63,64,65,66,67,68,69,70,71,72,73,74,75,76,77,78,79,80,81,82,83,84,85,86,87,88,89,90,91,92,93] **([50,53,66,68,77,78,81,85])**
Rib Fractures	Subtle cortical disruption, sclerosis, fracture line with callus formation, “pearls on a string” pattern in some cases	[7,13,40,46,91,92,93,94,95,96,97,98,99,100]
Fibrous Dysplasia	Ground-glass matrix, sclerotic border, expansile appearance with cortical thinning, cystic areas, intact bone borders	[7,12,13,40,42,93,101,102,103,104] **([101,104])**
Degenerative Changes/ Unspecific Benign	No significant morphological changes, non-specific lytic lesion with reactive changes, or normal bone tissue on CT	[13,92,93,105,106,107,108]
Paget Disease	Cortical thickening, irregular trabecular pattern, heterogeneous internal components	[92,93,109]
Osteoblastic Metastasis	Sclerotic lesion with osteoblastic features, often corresponding to tracer uptake	[58,78] **([78])**
Myelodysplastic Syndrome	No lytic or sclerotic changes, focal increased uptake mimicking metastasis	[12,110]
Fibrous Cortical Defect	Small hypoattenuating lesion with well-delimited sclerotic borders	[111]
Post-Traumatic Rib Lesion	Post-traumatic changes with faint uptake, no significant morphological changes	[96]

* The differential diagnoses are sorted by the number of reports found in the literature. ** References bolded are those with intense uptake or SUV greater than 10. The most probable diagnoses with intense uptake were prostate cancer metastases, fibrous dysplasia, and osteoblastic metastases from urothelial carcinoma.

**Table 2:** Review on original studies focusing on the incidence, diagnosis, and imaging characteristics of PSMA-avid rib lesions *.

First Author	Rate of Rib Lesions	Metastatic Rib Lesions	Isolated Rib (%)	Multiple Rib/Multifocal Uptake (%)	Rib SUV Range
**Studies with ^68^Ga-PSMA-11**					
Stoffels—2025 [7]	100% patients (inclusion criteria)	14.8% (8/54) **Cat I and III**	-	18.5% (10/54 patients had multiple rib lesions)	Mean: 2.90 (benign) and 4.8 (malignant)
Zacho—2020 [91]	6.25% (7/112) patients	No metastasis **Cat II**	100%	0%	-
Chen—2020 [40]	2.3% (111/4792) (62 of them had staging scans)	1.6% (1/62) **Cat I, III, IV**	100% (inclusion criteria)	-	Mean SUVmax: 2.21(malignant) 3.02(benign)
Sareni—2023 [132]	11.2% (20/179)	-	3.4% (6/179)	14/20 (70%) had additional bone sites (Multi focal)	Median SUVmax: 13.8 (all lesions)
Chiu—2020 [92]	33.9% (19/56)	79% (15/19) **Cat II**	-	-	-
Mainta—2024 [99]	30.6%	33.3% **Cat II**	-	-	Mean SUVmax: 4.6 (all lesions)
Simsek—2020 [100]	22.1% (25/113)	25/26 (96%) **Cat II**	-	(ribs + other sites): ~23.9%	-
Dancheva—2024 [133]	12.5% (5/40)	0% **Cat III/IV**	-	(symmetrical/multiple in ribs) ~23%	1.0–3.4 (bone lesions incl. ribs)
Shah—2025 [134]	4.9% (72/1480)	30.6% (22/72) **Cat I/III/IV**	Malignant: 40.9% (9/22); Benign: 51.6% (16/31)	(solitary focus; non-isolated: 59.1% with other mets)	Median range for lesions: Malignant: 4.31 (2.63–10.44); Benign: 2.18 (1.6–2.7)
**Studies with ^18^F-PSMA-1007**					
Luo—2024 [135]	37.3% (59/158)	11.8% (7/59) **Cat I and II**	-	-	-
Arnfield—2021 [13]	61.3% (122/199)	No metastasis **Cat II**	-	-	-
Benecke—2024 [136]	50% (115/230)	2% **Cat IV**	-	-	SUVmax range: 2.9–16.1(metastatic)
Grünig—2021 [12]	57.5%	-	-	-	Mean SUVmax: 3.8 (all lesions)
Bauckneht—2024 [137]	145/448(32.4%)	28.3% (41/145) **Cat II**	-	-	-
Hoberück—2023 [138]	58.7% (27/46)	-	-	-	-
Orevi—2022 [93]	40% (35/14)	No metastasis **Cat III**	11.8% solitary rib foci	Multifocal (including ribs and other sites): 29.4%	-
Seifert—2023 [139]	38.2% (13/34)	No metastasis **Cat II**	-		-
Fragkiadaki—2024 [140]	19.2% (20/104)	100% all metastatic **Cat II**	-	-	SUVmax range: 2.30–77.32 (all lesions)
Panagiotidis—2023 [141]	14%(26/186)	-	-	-	-
**Studies with ^18^F-DCFPyL**					
Woo—2025 [17]	100% (175/175)	47/175 (26.9%) **Cat II**	0.57% (1/175) for metastatic isolated rib lesions 54.9% (96/175) for single rib lesions with no other metastases	Multiple rib lesions only: 9.1% Multifocal (ribs + other sites): 24.0% (42/175)	Mean SUVmax and range: 1.8 (1.4, 2.6) (benign) 7.7 (2.7, 16.5) (malignant)
Ulaner—2022 [142]	11.7% (7/60)	57.1% (4/7) **Cat I**	100%	-	-
Phelps—2022 [143]	39/98(39.8%)	23% (9/39) **Cat I and II**	-	-	SUVmax range: (2.4 to 15.2)
Yin—2019 [144]	12/46(26.1%)	25% (3/12) **Cat IV**	-	-	Median SUVmax and range: 1.15, (0.85–1.89)

* For the diagnosis of metastatic lesions, the following 4 categories were considered: Category I: Biopsy/Histopathology: Confirmed through direct tissue sampling. Category II: Best Valuable Criteria: A combination of radiology, lab data, and other diagnostic factors. Category III: PSA Changes (i.e., PSA response following treatment or observation). Category IV: Other: Follow-up using imaging alone.

**Table 3:** Diagnostic factors and indicators of rib metastasis.

Factor	Description	Benign Characteristics	Metastatic Characteristics	Diagnosis Utility	Pitfalls
Radiotracer Uptake Intensity	Standardized uptake value maximum on PSMA PET/CT	Low-moderate (<10); often < liver uptake	High (>10); typically > liver uptake	Predicts malignancy likelihood; guides radioligand therapy	Tracer variability (^18^F-PSMA-1007 higher unspecific uptake); no standardized cutoff
Number of Lesions	Number of PSMA-avid rib lesions	Solitary (98% benign if isolated)	Multiple (97–98% associated with polymetastatic disease)	Indicates disease extent; solitary lesions often benign	Rare isolated metastases (2–3%); needs correlation with other sites
Location	Rib lesion site	Posterior ribs (often degenerative)	Anterior/lateral ribs (higher malignancy risk)	Guides biopsy prioritization	Limited data on site-specific malignancy rates
CT Morphology or Bone Scan	Structural features on CT (lytic or sclerotic)	Normal or degenerative	Lytic or sclerotic lesions; cortical disruption	Enhances specificity when combined with PSMA uptake	Non-specific findings
Patient-Specific Factors	Patient-specific factors (such as PSA and Gleason score)	Low PSA (<10 ng/mL); Gleason <7	High PSA (>20 ng/mL); Gleason ≥8	Contextualizes imaging; high-risk features suggest metastases	Non-specific; overlap in early disease
Changes Over Time	Longitudinal changes on serial PSMA PET/	Stable SUVmax, no new lesions; often degenerative or post-traumatic	Increasing SUVmax (>20% rise), new lesions, or morphological progression	Indicates disease progression; guides therapy adjustment	Requires serial imaging; limited data on optimal intervals; benign changes (such as healing fractures) may mimic progression
Tracer Type	PSMA ligand used (^18^F-PSMA-1007 vs. ^68^Ga-PSMA-11)	^18^F-PSMA-1007: higher unspecific bone uptake (20% equivocal)	^68^Ga-PSMA-11: lower unspecific uptake; higher specificity	Influences diagnostic accuracy; ^68^Ga-PSMA-11 preferred for rib lesions	^18^F-PSMA-1007 increases false positives; protocol variability

## Data Availability

No new data were created.

## Supplementary Materials

The following supporting information can be downloaded at: https://www.mdpi.com/article/10.3390/cancers17213404/s1; Table S1: Benign single rib lesions; Table S2: Benign Multiple Rib Lesions; Table S3: Malignant Single Rib Lesions; Table S4: Malignant Multiple Rib Lesions; Table S5: Equivocal Rib Lesions; Table S6: Complete List of References Containing Figures with PSMA-Avid Rib Lesions. References [164,165,166,167,168,169,170,171,172,173,174,175,176,177,178] are cited in the Supplementary Materials.
