# Supplementary material for: Fifty Shades of PSMA-Avid Rib Lesions: A Comprehensive Review
Source: Cancers (Basel). 2025 Oct 22;17(21):3404. doi: 10.3390/cancers17213404 (PMC12610356; doi:10.3390/cancers17213404)
Supplement: Supplementary file 1 [file cancers-17-03404-s001.zip › cancers-3914415-supplementary.pdf]

Supplementary Table S1: Benign Single Rib Lesions

Reference No.	First Author	Year	Lesion Diagnosis-Location	Lesion SUV_{max}	CT Scan Findings	PSMA Radioisotope
**(1)**	Reale	2018	Fibrous Dysplasia	**Intense**	Rib lesion with subtle cortical disruption, confirmed fibrous dysplasia	[^{68}Ga]Ga-PSMA-11
(2)	Plouznikoff	2019	Fibrous Dysplasia	Moderate	Expansile appearance with ground-glass and cystic areas, intact bone borders	[^{68}Ga]Ga-PSMA-11
(3)	De Coster	2017	Fibrous Dysplasia	-	Benign MRI features	[^{68}Ga]Ga-PSMA-11
(4)	Pontes	2023	Fibrous Cortical Defect	5.2	Small hypoattenuating lesion with well-delimited sclerotic borders	[^{68}Ga]Ga-PSMA-11
(5)	Kaewput	2022	Old Rib Fracture	3.1	Subtle sclerotic lesion	[^{18}F]F-PSMA-1007
(6)	Domínguez	2021	Focal uptake in the 6th bilateral costal arch	3.5	No significant changes in size; stable findings	[^{18}F]F-PSMA-1007
(7)	Zarbiv	2021	7th left rib	Slightly less uptake with [^{68}Ga]Ga-PSMA compared to [^{18}F]F-PSMA	No significant changes in interval scans	[^{18}F]F-PSMA-1007, [^{68}Ga]Ga-PSMA-11
(8)	Mapelli	2022	Bone lesion in posterior 10th rib	-	Non-significant uptake detected	[^{68}Ga]Ga-DOTA-RM2
(9)	Wondergem	2021	Right Rib Lesion (Patient 2)	-	Aspecific lytic lesion with reactive changes	[^{18}F]F-PSMA-1007
(9)	Wondergem	2021	Right Rib Lesion (Patient 3)	-	Normal bone tissue	[^{18}F]F-PSMA-1007
(10)	Anconina	2018	Lesion in 6th left rib (healing fracture)	-	Healing fracture in the 6th left rib	[^{68}Ga]Ga-PSMA-11
(11)	Arslan	2020	8th right rib lesion	-	No lytic or sclerotic changes; focal increased uptake mimicking metastasis	[^{68}Ga]Ga-PSMA-11
(12)	Ulaner	2022	2nd right rib lesion	6.9	Sclerosis seen on CT	[^{18}F]F-DCFPyL

(13)	Luo	2024	Right rib lesion (IBL)	4.3	No significant abnormality on CT	[^{18}F]F-PSMA-1007
(14)	König	2021	Rib fracture (non-displaced, with callus formation)	-	Fracture line visible with callus formation	[^{18}F]F-PSMA-1007
(14)	König	2021	4th right rib lesion	4.4	No corresponding morphological changes on CT	[^{18}F]F-PSMA-1007
(15)	Koehler	2023	Fractured rib	Low	Fractured rib with low PSMA expression	[^{68}Ga]Ga-PSMA-I&T
(16)	BEŞLİ	2019	6th left rib focus (bone scan)	Negative	Focal uptake on anterior part of 6th left rib	[^{68}Ga]Ga-PSMA-11
(17)	Luining	2021	3rd left rib (post-traumatic)	No abnormal uptake	Post-traumatic origin, faint uptake	Bone scintigraphy

Supplementary Table S2: Benign Multiple Rib Lesions

**Reference No.**	**First Author**	**Year**	**Lesion Diagnosis**	**Lesion SUV_{max}**	**CT Scan Findings**	**PSMA Radioisotope**
(18)	Jochumsen	2017	Rib Fractures	-	Rib fractures with "pearls on a string" pattern	[^{68}Ga]Ga-PSMA-11
(19)	Panagiotidis	2019	Rib Fractures	Moderately increased	Subtle cortical disruption and sclerosis in ribs (2nd right, 4th, and 5th left ribs)	[^{18}F]F-PSMA-1007
(20)	Blazak	2016	Paget Disease	-	Cortical thickening, irregular trabecular pattern (Paget disease)	[^{68}Ga]Ga-PSMA-11
**(21)**	Wang	2020	Fibrous Dysplasia	**Intense**	Expansive changes, ground-glass appearance, cortical erosion, diffuse sclerosis	[^{18}F]F-rhPSMA-7
(22)	Lenis	2022	Foci on 7th right rib and 10th left rib	None detected	No significant PSMA uptake; visible on [^{99m}Tc]Tc-MDP bone scan	[^{68}Ga]Ga-PSMA-11

(23)	Mainta	2024	PSMA-RADS-1 (Rib fractures)	2.9, 3	Consolidation on follow-up	$[^{68}Ga]$Ga-PSMA-11
(24)	de Galiza Barbosa	2020	Rib fractures	Mild uptake	Continuous rib fractures in CT	$[^{68}Ga]$Ga-PSMA-11
(25)	Afaq	2017	Degenerative rib changes (other ribs)	Low	Probable degenerative changes, no suspicious uptake ($[^{99m}Tc]$Tc-MDP scan)	$[^{99m}Tc]$Tc-MDP, $[^{68}Ga]$Ga-PSMA-11
(26)	Curcean	2022	False-positive rib lesions	-	PSMA PET/CT and PSMA MIP show two small areas of rib uptake	-
(27)	Urso	2023	Rib lesions (likely UBU)	Stable in number, size, and uptake intensity	No structural changes detected in CT images	$[^{18}F]$F-PSMA-1007
(28)	von Stauffenberg	2024	Unspecific bone uptake	-	No morphological correlates	$[^{18}F]$F-PSMA-1007

Supplementary Table S3: Malignant Single Rib Lesions

Reference No.	First Author	Year	Lesion Diagnosis	Lesion SUV_{max}	CT Scan Findings	PSMA Radioisotope
(29)	Trent	2020	Prostate carcinoma metastasis	-	Lytic lesion in the 7th left rib with aggressive appearance, initially misinterpreted as a rib fracture	-
(30)	Kishore	2012	Prostate carcinoma metastasis	-	Focal lesion in the 2nd right rib; rapid enlargement of the prostate on MRI and CT scan	-
(31)	Chen	2022	Metastatic bone lesion on 5th left rib	5.1	Not detected on CT or bone scan	$[^{68}Ga]$Ga-PSMA-11
**(32)**	Elkanawaty	2021	9th left rib lesion	**10.4**	Not detected on CECT	$[^{68}Ga]$Ga-PSMA-11
(33)	Mena	2019	Posterior 5th right rib lesion	-	No definitive abnormality on CT	$[^{18}F]$F-DCFPyL

(34)	Cytawa	2020	5th right rib	-	No anatomical correlate	[68Ga]Ga-PSMA-I&T
(35)	Li	2022	6th left rib lesion	2.97	No obvious bone destruction on CT	[99mTc]Tc-HYNIC-PSMA
(35)	Li	2022	8th right rib lesion	8.63	Bone destruction observed on CT	[99mTc]Tc-HYNIC-PSMA
**(36)**	Masselli	2024	7th left rib	**13**	No changes on initial CT; post-RT myositis on follow-up	[68Ga]Ga-PSMA-11
(37)	Liu	2019	Bone metastasis	4.37	Single bone metastasis	[18F]F-PSMA-1007
(37)	Liu	2019	Bone metastasis	-	Visible on CT and PET/CT	[68Ga]Ga-PSMA-11
(38)	Lütje	2017	8th right rib lesion	-	Bone lesion identified in CT component only	[68Ga]Ga-PSMA-11
(39)	Zieniewicz	2020	Metastatic rib lesion (6th right rib)	2.2	Bone lesion at the 6th right rib, consistent with metastasis	[68Ga]Ga-PSMA-11
(40)	Simsek	2019	Sclerotic metastasis in 5th right rib	-	Sclerotic metastasis in the 5th right rib	[68Ga]Ga-PSMA-11
(41)	Yadav	2020	5th left rib (residual)	Decreased	Decreased size, number, and PSMA uptake (interim scan)	[68Ga]Ga-PSMA-11
(42)	Muzahir	2023	Osteoblastic rib lesion metastasis	Mild	Osteoblastic lesion (axial CT, chest)	[18F]F-PyL
(42)	Muzahir	2023	Right-sided rib uptake	Mild	Mild uptake in right rib (axial CT, chest)	[18F]F-PyL
(43)	Jafari	2024	8th right rib lesion	-	Suspicious focal uptake without morphological changes on CT	[68Ga]Ga-PSMA-11
(44)	Herrmann	2015	Rib metastasis	Increased	Uptake detected on sequential scans (early to 4 hours)	[68Ga]Ga-PSMA-11
(45)	Heinzel	2019	Right rib lesion	Δ SUV: -61%	PET showed decreased uptake in right rib lesion, false-positive	[68Ga]Ga-PSMA-11
(46)	Seniaray	2020	5th left rib lesion	-	PSMA-avid sclerotic lesion	[68Ga]Ga-PSMA-11
(47)	Wondergem	2021	Rib lesion	-	Lytic lesion	[18F]F-DCFPyL

(48)	Raju	2020	Rib lesion	Mild	Not specified; treated with radiotherapy	[^{18}F]F-PSMA-1007, [^{18}F]F-NaF
(49)	Stasiak	2023	6th right rib lesion	8.8	Small sclerotic lesion on CT	[^{68}Ga]Ga-PSMA-11
(50)	Kooraki	2023	Right-side rib lesion	Faint	No CT correlation, no morphological changes on CT	[^{68}Ga]Ga-PSMA-11
(50)	Kooraki	2023	4th right rib lesion	Focal	Focal sclerotic lesion on CT, compatible with metastasis	[^{18}F]F-DCFPyL
(50)	Kooraki	2023	2nd left rib lesion	-	Very faint sclerosis on CT	[^{18}F]F-DCFPyL
**(51)**	Rowe	2017	Rib lesion	**Intense**	No morphological changes seen on CT; focal uptake visible on axial PET/CT	[^{18}F]F-DCFPyL
(52)	Talbot	2019	3rd left rib focus	2.0	Discrete focus on rib	[^{68}Ga]Ga-PSMA-11
**(53)**	Vetrone	2024	4th right rib (dorsal tract)	**Intense**	Bone thickening at the dorsal tract of the rib	[^{68}Ga]Ga-PSMA-11
(54)	Mena	2020	5th right rib focus	-	Focus on 5th right rib with confirmed bone metastasis	[^{18}F]F-DCFPyL
(55)	Baum	2017	Skeletal metastasis (3rd left rib)	-	Uptake in skeletal metastasis near the sternocostal junction	[^{152}Tb]Tb-DOTATOC
(56)	Witkowska-Patena	2019	Bone metastasis in 5th right rib	2.9	Slight opacities in the 5th right rib	[^{18}F]F-PSMA-1007
(57)	Belue	2024	1st left rib lesion (additional metastasis)	-	Segmented and classified as metastatic by 3DAISeg and 3DAIClass	[^{68}Ga]Ga-PSMA-11
(58)	Rowe	2020	Bone lesion in 8th right rib	-	Lesion suggestive of metastatic disease in 8th right rib	[^{18}F]F-DCFPyL
(59)	Smith	2019	Sclerotic bone lesion at posterior 4th right rib	-	Small DCFPyL-avid sclerotic bone lesion at posterior 4th right rib	[^{18}F]F-DCFPyL
(60)	Lucas	2023	1st left rib metastasis	-	1st left rib metastasis visualized only on [^{18}F]F-DCFPyL scan	[^{18}F]F-DCFPyL
(60)	Lucas	2023	4th right rib metastasis	-	4th right rib metastasis	[^{18}F]F-DCFPyL

(61)	Долгушин	2018	Right rib metastasis	-	No specific lesion on CT (likely due to bone sclerosis)	[^{18}F]F-PSMA-1007

Supplementary Table S4: Malignant Multiple Rib Lesions

Reference No.	First Author	Year	Lesion Diagnosis	Lesion SUV_{max}	CT Scan Findings	PSMA Radioisotope
**(62)**	Zhao	2022	Urothelial carcinoma with bone metastases	**12.4**	Isodensity to hyperdensity, multiple foci in ribs and sternum	[^{68}Ga]Ga-PSMA-11
**(63)**	Grawe	2023	Osteoblastic metastasis	**Intense**	Osteoblastic metastasis with suggestive tracer uptake	[^{18}F]F-PSMA-1007
(5)	Kaewput	2022	Metastases (prostate cancer)	-	Mixed osteolytic and blastic metastases	[^{18}F]F-PSMA-1007
(64)	Werner	2018	6th right rib metastasis; right iliac LN indeterminate	-	CT revealed findings corresponding to 6th right rib metastasis; mild radiotracer uptake in right iliac LN	[^{18}F]F-DCFPyL
(65)	Petersen	2017	5th right rib, 3rd left rib	-	Mixed osteosclerotic and osteolytic lesions	[^{68}Ga]Ga-PSMA-11, [^{18}F]F-NaF
**(66)**	Tatar	2023	Bone metastases (6th & 8th ribs)	**Intense**	Intense PSMA uptake in 6th & 8th ribs	-
(67)	Alipour	2019	10th left rib (posterior)	High	Visible on PSMA PET & bone scan	[^{68}Ga]Ga-PSMA-11
(67)	Alipour	2019	4th right rib	High	Avid uptake on PSMA PET	[^{68}Ga]Ga-PSMA-11
(68)	Regula	2022	Bone lesions (sclerotic)	Reduced	Sclerotic lesions on ribs	[^{68}Ga]Ga-PSMA-11
(69)	Filippov	2020	Metastatic prostate cancer	High	FDG PET: nonspecific activity; [^{68}Ga]Ga-PSMA: focal uptake in skeletal structures (e.g., clavicle, ribs, vertebrae)	[^{68}Ga]Ga-PSMA-11

(70)	Al-Ibraheem	2023	Prostate and colorectal cancer	High	[^{68}Ga]Ga-PSMA: prostate, seminal vesicles, internal iliac lymph nodes, ribs, T7, left femur	[^{68}Ga]Ga-PSMA-11
**(71)**	Werner	2023	Focal intense uptake in two ribs	**Intense**	Sclerotic changes in corresponding ribs	[^{18}F]F-PSMA-1007
(72)	Loeff	2024	Ribs lesions	-	Low-level uptake in ribs (unspecific findings)	[^{18}F]F-PSMA-1007
(27)	Urso	2023	Rib lesions (PCa metastasis)	Focal uptakes	No specific structural changes observed, only uptake seen in various regions	[^{68}Ga]Ga-PSMA-11
(73)	Avul	2019	Rib lesion (PCa metastasis)	Varies	Presence of metastasis in the right side of ribs	[^{68}Ga]Ga-PSMA-11
(74)	Tuncel	2020	8th right rib	Increased	Foci of increased radiotracer uptake on bone scan	[^{68}Ga]Ga-PSMA-11
(75)	Jadvar	2022	Right ribs (additional metastases)	-	Metastatic lesions in right ribs	[^{68}Ga]Ga-PSMA-11
(76)	Pouliot	2024	Metastatic CRPC (mCRPC) in bone (rib)	-	Metastases in bones (including rib)	[^{68}Ga]Ga-PSMA-617
(77)	Abdel-Aty	2024	Bone metastases (possible rib involvement)	-	Non-specific degenerative changes	[^{68}Ga]Ga-PSMA-11

Supplementary Table S5: Equivocal Rib Lesions

Reference No.	First Author	Year	Lesion Diagnosis	Single/Multiple	Lesion SUV_{max}	CT Scan Findings	PSMA Radioisotope
(78)	Werner	2018	Left-sided rib lesion (PSMA-RADS-3B)	Single	Mild	No pathological enlargement observed	[^{18}F]F-DCFPyL
(79)	Hoffman	2023	Rib lesion	Single	-	No apparent lesion on CT	[^{68}Ga]Ga-PSMA-11
(36)	Masselli	2024	3rd right rib	Single	2	No clear morphologic correlation initially	[^{68}Ga]Ga-PSMA-11

(80)	Vargas-Ahumada	2023	5th right rib uptake	Single	-	No evidence of blastic lesions	$[^{18}F]$F-PSMA-1007
(71)	Werner	2023	Focal low uptake in rib	Single	Low	No corresponding lesion in axial bone window on CT	$[^{18}F]$F-PSMA-1007

Supplementary Table S6: Complete List of References Containing Figures with PSMA-Avid Rib Lesions

First author	Diagnosis	year	Journal	DOI	Fig. number
Arnfield EG	Benign - False positive	2021	European Journal of Nuclear Medicine and Molecular Imaging	10.1007/s00259-021-05456-3	4
Panagiotidis E	Benign – Rib fracture	2019	Clinical Nuclear Medicine	10.1097/RLU.0000000000002354	1
Zacho HD	Benign	2020	EJNMMI Research	10.1186/s13550-020-00618-0	3
Stoffels M	Benign - False positive	2025	Nuclear Medicine Communications	10.1097/MNM.0000000000001919	2
Stoffels M	Benign	2025	Nuclear Medicine Communications	10.1097/MNM.0000000000001919	3
Stoffels M	Metastasis	2025	Nuclear Medicine Communications	10.1097/MNM.0000000000001919	4
Houshmand S	Benign – Rib Fracture	2023	Abdominal Radiology	10.1007/s00261-023-04002-z	7
Phelps TE	Benign	2023	Journal of Nuclear Medicine	10.2967/jnumed.122.264334	3
De Coster L	Benign - Fibrous dysplasia	2017	European Journal of Nuclear Medicine and Molecular Imaging	10.1007/s00259-017-3712-6	Main image
Husseini JS	Metastasis	2021	European Journal of Nuclear Medicine and Molecular Imaging	10.1007/s00259-021-05198-2	6
Oldan JD	Metastasis	2024	The Cancer Journal	10.1097/PPO.0000000000000718	3

Sheikhbahaei S	Benign	2017	European Journal of Nuclear Medicine and Molecular Imaging	10.1007/s00259-017-3780-7	7
Reale ML	Benign - Fibrous Dysplasia	2019	Clinical Nuclear Medicine	10.1097/RLU.0000000000002546	1
Baucknehst M	Benign	2024	Journal of Nuclear Medicine	10.2967/jnumed.124.267751	4
Chiu LW	Metastasis	2020	Journal of Nuclear Medicine	10.2967/jnumed.119.241174	3
Pontes ÍC	Benign	2023	Radiologia Brasileira	10.1590/0100-3984.2023.0003	7
Shetty D	Benign – Healing fracture	2018	Tomography	10.18383/j.tom.2018.00021	7
Grawe F	Metastasis – RADS I	2024	European Radiology	10.1007/s00330-023-10083-7	3
Wang H	Benign – Fibrous Dysplasia	2020	European Journal of Nuclear Medicine and Molecular Imaging	10.1007/s00259-020-04751-9	Main image
Orevi M	Benign – Fibrous Dysplasia	2022	Frontiers in Surgery	10.3389/fsurg.2022.943760	2
Gafita A	Metastasis	2022	American Society of Clinical Oncology Educational Book	10.1200/EDBK_350946	1
Werner RA	Benign	2018	Annals of Nuclear Medicine	10.1007/s12149-018-1291-7	4
Plouznikoff N	Benign – Fibrous Dysplasia	2019	Clinical Nuclear Medicine	10.1097/RLU.0000000000002609	Main image
Jochumsen MR	Benign – Rib fracture	2018	Clinical Nuclear Medicine	10.1097/RLU.0000000000001871	Main image
Voter AF	Benign – Fibrous Dysplasia	2024	Seminars in Nuclear Medicine	10.1053/j.semnuclmed.2023.11.001	5
Chen MY	Metastasis	2020	BJU International	10.1111/bju.15152	1

Kaewput C	Benign	2022	Journal of Clinical Medicine	10.3390/jcm11102738	4
Al-Ibraheem A	Benign	2023	Nuclear Medicine and Molecular Imaging	10.1007/s13139-023-00812-7	2
Curcean A	Benign	2022	Clinical Radiology	10.1016/j.crad.2021.09.005	4
Yadav MP	Metastasis	2020	Clinical Nuclear Medicine	10.1097/RLU.0000000000002833	2
Jafari E	Metastasis	2024	Clinical and Translational Imaging	10.1007/s40336-024-00668-7	7
Herrmann K	Metastasis	2015	Journal of Nuclear Medicine	10.2967/jnumed.114.148130	2
Al-Ibraheem A	Metastasis	2023	Nuclear Medicine and Molecular Imaging	10.1007/s13139-023-00812-7	2
Afaq A	Metastasis	2017	International Urology and Nephrology	10.1007/s11255-017-1541-y	2
Kunikowska J	Metastasis	2021	European Journal of Nuclear Medicine and Molecular Imaging	10.1007/s00259-020-05017-0	1
Werner RA	Metastasis	2023	European Urology	10.1016/j.eururo.2023.06.008	9
Lee SJ	Metastasis	2023	Exon Publications	10.36255/molecular-targeted-therapy-for-prostate-cancer	2
Filippov A	Metastasis	2020	Journal of Thoracic Disease	10.21037/jtd-2019-pitd-13	1
Arslan İ	Malignant - Myelodysplastic Syndrome	2020	Clinical Nuclear Medicine	10.1097/RLU.0000000000002964	1
Regula N	Metastasis	2022	European Journal of Hybrid Imaging	10.1186/s41824-022-00127-4	4
Avul AR	Metastasis	2019	Turkish Medical Student Journal	https://dergipark.org.tr/en/pub/tmsj/issue/43969/541685	1
Urso L	Benign	2023	Journal of Clinical Medicine	10.3390/jcm12227130	1
Rathke H	Metastasis	2018	Journal of Nuclear Medicine	10.2967/jnumed.117.200220	4
Wondergem M	Metastasis	2021	European Journal of Nuclear Medicine and Molecular Imaging	10.1007/s00259-020-04782-2	3
Luo L	Equivocal	2024	Clinical Radiology	10.1016/j.crad.2023.12.008	2
Ulaner GA	Metastasis	2022	Radiology	10.1148/radiol.220218	2

Seniaray N	Metastasis	2020	Indian Journal of Urology	10.4103/iju.IJU_275_19	3
Rowe SP	Metastasis	2017	PET Clinics	10.1016/j.cpet.2017.02.006	2
Koehler D	Benign – Rib Fracture	2023	RöFo-Fortschritte auf dem Gebiet der Röntgenstrahlen	10.1055/a-2088-9543	1
Tuncel M	Metastasis	2020	Clinical Nuclear Medicine	10.1097/RLU.0000000000003126	3
Vetrone L	Metastasis	2024	Seminars in Nuclear Medicine	10.1053/j.semnuclmed.2023.06.004	4
Mena E	Metastasis	2020	Journal of Nuclear Medicine	10.1053/j.semnuclmed.2019.02.001	5
Jadvar H	Metastasis	2022	Journal of Nuclear Medicine	10.2967/jnumed.121.263124	1
Belue MJ	Metastasis	2024	Academic Radiology	10.1016/j.acra.2024.01.009	6
Khojasteh E	Metastasis	2023	Journal of Bone Oncology	10.1016/j.jbo.2023.100477	4
Dolgushin	Metastasis	2018	Онкоурология	10.17650/1726-9776-2018-14-3-134-138	2
Vargas-Ahumada JE	Benign	2023	Cancers	10.3390/cancers15245824	4
Lütje S	Metastasis	2017	Clinical Nuclear Medicine	10.1097/RLU.0000000000001454	4
Blazak JK	Benign – Paget	2016	Clinical Nuclear Medicine	10.1097/RLU.0000000000001296	Main image
Grünig H	Benign	2021	European Journal of Nuclear Medicine and Molecular Imaging	10.1007/s00259-021-05424-x	8
Masselli G	Metastasis	2024	Diagnostics	10.3390/diagnostics14121291	2
Masselli G	Metastasis – RADS II	2024	Diagnostics	10.3390/diagnostics14121291	6
Alipour R	Metastasis	2019	Therapeutic Advances in Medical Oncology	10.1177/1758835919876828	6
Malan N	Benign – Rib Fracture	2022	Frontiers in Nuclear Medicine	10.3389/fnume.2022.825512	6
Mainta IC	Benign – Rib Fracture	2024	Journal of Nuclear Medicine	10.2967/jnumed.124.267899	2
Li B	Metastasis	2022	Frontiers in Oncology	10.3389/fonc.2022.1072437	3
Tatar G	Metastasis	2023	Clinical Nuclear Medicine	10.1097/RLU.0000000000004522	4

Zarbiv Y	Benign	2021	Cancer Reports	10.1002/cnr2.1386	3
Petersen LJ	Metastasis	2017	Molecular and Clinical Oncology	10.3892/mco.2017.1280	1
Cytawa W	Benign	2020	European Journal of Nuclear Medicine and Molecular Imaging	10.1007/s00259-019-04524-z	7
Mena E	Metastasis	2019	Seminars in Nuclear Medicine	10.1053/j.semnuclmed.2019.02.001	5

References:

1. Reale ML, Buttigliero C, Tucci M, Giardino R, Poti C. 68Ga-PSMA uptake in fibrous dysplasia. Clinical nuclear medicine. 2019;44(6):e396-e7.
2. Plouznikoff N, Garcia C, Artigas C, Entezari K, Flamen P. Heterogeneity of 68Ga-PSMA PET/CT uptake in fibrous dysplasia. Clinical Nuclear Medicine. 2019;44(10):e593-e4.
3. De Coster L, Sciot R, Everaerts W, Gheysens O, Verscuren R, Deroose CM, et al. Fibrous dysplasia mimicking bone metastasis on 68 GA-PSMA PET/MRI. European journal of nuclear medicine and molecular imaging. 2017;44:1607-8.
4. Pontes ÍCdM, Souza AR, Fonseca EKUN, Osawa A, Baroni RH, Castro AdAe. Musculoskeletal pitfalls in 68Ga-PSMA PET/CT. Radiologia Brasileira. 2023;56(4):220-5.
5. Kaewput C, Vinjamuri S. Update of PSMA theranostics in prostate cancer: current applications and future trends. Journal of Clinical Medicine. 2022;11(10):2738.
6. Domínguez JG, de León JMSP, Cortés JLC. PET/CT with 18F-PSMA in Patients with Prostate Cancer, Review of the Initial Experience. Open Journal of Urology. 2021;11(4):158-75.
7. Zarbiv Y, Peerless Y, Wygoda M, Orevi M, Meir K, Gofrit ON, et al. Real-world Israeli single institution experience with PET-PSMA for staging of patients with clinically staged localized prostate carcinoma. Cancer Reports. 2021;4(5):e1386.
8. Mapelli P, Ghezzo S, Samanes Gajate AM, Preza E, Palmisano A, Cucchiara V, et al. 68Ga-PSMA and 68Ga-DOTA-RM2 PET/MRI in recurrent prostate cancer: diagnostic performance and association with clinical and histopathological data. Cancers. 2022;14(2):334.
9. Wondergem M, van der Zant FM, Broos WA, Knol RJ. Matched-pair comparison of 18F-DCFPyL PET/CT and 18F-PSMA-1007 PET/CT in 240 prostate cancer patients: interreader agreement and lesion detection rate of suspected lesions. Journal of Nuclear Medicine. 2021;62(10):1422-9.
10. Anconina R, Hod N, Levin D, Kazap DE, Lantsberg S. Incidental detection of metastatic malignant melanoma on 68Ga–prostate-specific membrane antigen PET/CT imaging: correlative imaging with FDG PET/CT and review of the literature. Clinical Nuclear Medicine. 2018;43(3):204-6.
11. Arslan İ, Demirci E, Özkara S, Taşdelen N, Selçuk NA. Myelodysplastic syndrome presenting with diffuse bone marrow uptake on 68Ga-PSMA PET/CT. Clinical Nuclear Medicine. 2020;45(4):330-3.
12. Ulaner GA, Thomsen B, Bassett J, Torrey R, Cox C, Lin K, et al. 18F-DCFPyL PET/CT for initially diagnosed and biochemically recurrent prostate cancer: prospective trial with pathologic confirmation. Radiology. 2022;305(2):419-28.
13. Luo L, Wang Z, Wang X, Gao J, Zheng A, Duan X. Fluorine-18 prostate-specific membrane antigen-1007-avid indeterminate bone lesions in prostate cancer: clinical and PET/CT features to predict outcomes and prognosis. Clinical Radiology. 2024;79(5):346-53.
14. König MM. PSMA-Liganden PET/CT bei Patienten mit biochemischem Rezidiv nach radikaler Prostatektomie: Matched-Pair Vergleich von 68Ga-PSMA-11 und 18F-PSMA-1007: Technische Universität München; 2021.
15. Koehler D, Berliner C, Shenas F, Karimzadeh A, Apostolova I, Klutmann S, et al., editors. PSMA hybrid imaging in prostate cancer–current applications and perspectives. RöFo-Fortschritte auf dem Gebiet der Röntgenstrahlen und der bildgebenden Verfahren; 2023: Georg Thieme Verlag KG.
16. BEŞLİ RLU, SAĞER MS, Akgün E, Asa S, Şahin OE, Demirdağ Ç, et al. Comparison of Ga-68 PSMA positron emission tomography/computerized tomography with Tc-99m MDP bone scan in prostate cancer patients. Turkish journal of medical sciences. 2019;49(1):301-10.
17. Luining WI, Meijer D, Dahele MR, Vis AN, Oprea-Lager DE. Nuclear imaging for bone metastases in prostate cancer: the emergence of modern techniques using novel radiotracers. Diagnostics. 2021;11(1):117.

18. Jochumsen MR, Dias AH, Bouchelouche K. Benign traumatic rib fracture: a potential pitfall on 68Ga–prostate-specific membrane antigen PET/CT for prostate cancer. Clinical nuclear medicine. 2018;43(1):38-40.
19. Panagiotidis E, Paschali A, Giannoula E, Chatzipavlidou V. Rib fractures mimicking bone metastases in 18F-PSMA-1007 PET/CT for prostate cancer. Clinical nuclear medicine. 2019;44(1):e46-e8.
20. Blazak JK, Thomas P. Paget disease: a potential pitfall in PSMA PET for prostate cancer. Clinical nuclear medicine. 2016;41(9):699-700.
21. Wang H, Eiber M, Langbein T. A rare case of polyostotic fibrous dysplasia detected on 18 F-rhPSMA-7 PET/CT. European Journal of Nuclear Medicine and Molecular Imaging. 2020;47:2927-9.
22. Lenis AT, Pooli A, Lec PM, Sadun TY, Johnson DC, Lebacle C, et al. Prostate-specific membrane antigen positron emission tomography/computed tomography compared with conventional imaging for initial staging of treatment-naïve intermediate-and high-risk prostate cancer: a retrospective single-center study. European Urology Oncology. 2022;5(5):544-52.
23. Mainta IC, Neroladaki A, Wolf NB, Benamran D, Boudabbous S, Zilli T, et al. [68Ga] Ga-PSMA-11 PET and Prostate Cancer Bone Metastases: Diagnostic Performance of Available Standardized Criteria. Journal of Nuclear Medicine. 2024;65(9):1376-82.
24. de Galiza Barbosa F, Queiroz MA, Nunes RF, Costa LB, Zaniboni EC, Marin JFG, et al. Nonprostatic diseases on PSMA PET imaging: a spectrum of benign and malignant findings. Cancer Imaging. 2020;20:1-23.
25. Afaq A, Batura D, Bomanji J. New frontiers in prostate cancer imaging: clinical utility of prostate-specific membrane antigen positron emission tomography. International urology and nephrology. 2017;49:803-10.
26. Curcean A, Curcean S, Rescigno P, Ap Dafydd D, Tree A, Reid A, et al. Imaging features of the evolving patterns of metastatic prostate cancer. Clinical Radiology. 2022;77(2):88-95.
27. Urso L, Filippi L, Castello A, Marzola MC, Bartolomei M, Cittanti C, et al. PSMA PET/CT in castration-resistant prostate cancer: myth or reality? Journal of Clinical Medicine. 2023;12(22):7130.
28. von Stauffenberg F, Poyet C, Beintner-Skawran S, Maurer A, Schmid FA. Current clinical applications of PSMA-PET for prostate cancer diagnosis, staging, and treatment. Cancers. 2024;16(24):4263.
29. Trent SM, Krumme JW, Henshaw RM. Metastatic prostate carcinoma: A rare presentation initially misdiagnosed as a rib fracture. Radiology Case Reports. 2020;15(10):1795-8.
30. Kishore T, Shetty A, Tharun B, Joycee M. A rare case of prostatic malignancy. Indian Journal of Medical Sciences. 2012;66(7/8):189.
31. Chen J, Qi L, Tang Y, Tang G, Gan Y, Cai Y. Current role of prostate-specific membrane antigen-based imaging and radioligand therapy in castration-resistant prostate cancer. Frontiers in Cell and Developmental Biology. 2022;10:958180.
32. Elkanawaty M, Farouk H, Abdel Kawi M. Added Value of 68Gallium-PSMA PET/CT Over Contrast Enhanced CT in Prostate Cancer Patients. Benha Medical Journal. 2021.
33. Mena E, Lindenberg LM, Choyke PL, editors. New targets for PET molecular imaging of prostate cancer. Seminars in Nuclear Medicine; 2019: Elsevier.
34. Cytawa W, Seitz AK, Kircher S, Fukushima K, Tran-Gia J, Schirbel A, et al. 68 Ga-PSMA I&T PET/CT for primary staging of prostate cancer. European journal of nuclear medicine and molecular imaging. 2020;47:168-77.
35. Li B, Duan L, Shi J, Han Y, Wei W, Cheng X, et al. Diagnostic performance of 99mTc-HYNIC-PSMA SPECT/CT for biochemically recurrent prostate cancer after radical prostatectomy. Frontiers in Oncology. 2022;12:1072437.
36. Masselli G, Sollaku S, De Angelis C, Polettini E, Gualdi G, Casciani E. 68Ga-PSMA PET/CT in Recurrent Prostate Cancer after Radical Prostatectomy Using PSMA-RADS Version 2.0. Diagnostics. 2024;14(12):1291.

37. Liu T, Liu C, Xu X, Liu F, Guo X, Li N, et al. Preclinical evaluation and pilot clinical study of Al18F-PSMA-BCH for prostate cancer PET imaging. Journal of Nuclear Medicine. 2019;60(9):1284-92.
38. Lütje S, Gomez B, Cohnen J, Umutlu L, Gotthardt M, Poeppel TD, et al. Imaging of prostate-specific membrane antigen expression in metastatic differentiated thyroid cancer using 68Ga-HBED-CC-PSMA PET/CT. Clinical nuclear medicine. 2017;42(1):20-5.
39. Zieniewicz K, Królicki L. Jolanta Kunikowska1 & Bartosz Cieślak2 & Beata Gierej3 & Waldemar Patkowski4 & Leszek Kraj5, 6 & Marcin Kotulski4 &. 2020.
40. Simsek DH, Sanli Y, Kuyumcu S, Engin MN, Buyukkaya F, Demirci E. Clinical impact of lower-limb imaging in 68Ga-PSMA PET/CT for patients with prostate cancer. Journal of Nuclear Medicine Technology. 2019;47(3):233-7.
41. Yadav MP, Ballal S, Bal C, Sahoo RK, Damle NA, Tripathi M, et al. Efficacy and safety of 177Lu-PSMA-617 radioligand therapy in metastatic castration-resistant prostate cancer patients. Clinical nuclear medicine. 2020;45(1):19-31.
42. Muzahir S, Grady EE, Lee SJ. Molecular Targeted Radionuclide Therapy for Prostate Cancer. Exon Publications. 2023:171-85.
43. Jafari E, Dadgar H, Zarei A, Samimi R, Manafi-Farid R, Divband G, et al. Correction: The role of [68Ga] Ga-PSMA PET/CT in primary staging of newly diagnosed prostate cancer: predictive value of PET-derived parameters for risk stratification through machine learning. Clinical and Translational Imaging. 2024:1-.
44. Herrmann K, Bluemel C, Weineisen M, Schottelius M, Wester H-J, Czernin J, et al. Biodistribution and radiation dosimetry for a probe targeting prostate-specific membrane antigen for imaging and therapy. Journal of Nuclear Medicine. 2015;56(6):855-61.
45. Heinzel A, Boghos D, Mottaghy FM, Gaertner F, Essler M, von Mallek D, et al. 68 Ga-PSMA PET/CT for monitoring response to 177 Lu-PSMA-617 radioligand therapy in patients with metastatic castration-resistant prostate cancer. European Journal of Nuclear Medicine and Molecular Imaging. 2019;46:1054-62.
46. Seniaray N, Verma R, Khanna S, Belho E, Pruthi A, Mahajan H. Localization and restaging of carcinoma prostate by 68Gallium prostate-specific membrane antigen positron emission tomography computed tomography in patients with biochemical recurrence. Indian Journal of Urology. 2020;36(3):191-9.
47. Wondergem M, Van Der Zant F, Broos W, Roeleveld T, Donker R, Ten Oever D, et al. 18 F-DCFPyL PET/CT for primary staging in 160 high-risk prostate cancer patients; metastasis detection rate, influence on clinical management and preliminary results of treatment efficacy. European Journal of Nuclear Medicine and Molecular Imaging. 2021;48:521-31.
48. Raju S, Sharma A, Patel C, Sahoo R, Das CJ, Kumar S, et al. Is there a utility of adding skeletal imaging to 68-Ga-prostate-specific membrane antigen-PET/computed tomography in initial staging of patients with high-risk prostate cancer? Nuclear Medicine Communications. 2020;41(11):1183-8.
49. Stasiak CES, Cardillo A, Almeida SAd, Rodrigues RS, Castro PHRd, Parente DB. Preoperative evaluation of prostate cancer by 68 Ga-PMSA positron emission tomography/computed tomography: comparison with magnetic resonance imaging and with histopathological findings. Radiologia Brasileira. 2023;56:171-8.
50. Kooraki S, Jadvar H. Correlative Approach to Prostate Imaging. Radiology-Nuclear Medicine Diagnostic Imaging: A Correlative Approach. 2023:533-53.
51. Rowe SP, Gorin MA, Pomper MG. Imaging of prostate-specific membrane antigen using [18F] DCFPyL. PET clinics. 2017;12(3):289-96.
52. Talbot J-N, Aveline C, Zhang-Yin J, Nataf V, Rusu T, Balogova S, et al. Imagerie du cancer de la prostate oligométastatique, le point de vue du médecin nucléaire. Médecine Nucléaire. 2019;43(2):227-35.

53. Vetrone L, Fortunati E, Castellucci P, Fanti S, editors. Future imaging of prostate cancer: do we need more than PSMA PET/CT? Seminars in Nuclear Medicine; 2024: Elsevier.
54. Mena E, Lindenberg ML, Turkbey IB, Shih JH, Harmon SA, Lim I, et al. 18F-DCFPyL PET/CT imaging in patients with biochemically recurrent prostate cancer after primary local therapy. Journal of Nuclear Medicine. 2020;61(6):881-9.
55. Baum RP, Singh A, Benešová M, Vermeulen C, Gnesin S, Köster U, et al. Clinical evaluation of the radiolanthanide terbium-152: first-in-human PET/CT with 152 Tb-DOTATOC. Dalton transactions. 2017;46(42):14638-46.
56. Witkowska-Patena E, Giżewska A, Dziuk M, Miśko J, Budzyńska A, Walęcka-Mazur A. Head-to-head comparison of 18F-prostate-specific membrane antigen-1007 and 18F-fluorocholine PET/CT in biochemically relapsed prostate cancer. Clinical nuclear medicine. 2019;44(12):e629-e33.
57. Belue MJ, Harmon SA, Yang D, An JY, Gaur S, Law YM, et al. Deep Learning-Based Detection and Classification of Bone Lesions on Staging Computed Tomography in Prostate Cancer: A Development Study. Academic radiology. 2024;31(6):2424-33.
58. Rowe SP, Li X, Trock BJ, Werner RA, Frey S, DiGianvittorio M, et al. Prospective comparison of PET imaging with PSMA-targeted 18F-DCFPyL versus Na18F for bone lesion detection in patients with metastatic prostate cancer. Journal of Nuclear Medicine. 2020;61(2):183-8.
59. Smith CP, Laucis A, Harmon S, Mena E, Lindenberg L, Choyke PL, et al. Novel imaging in detection of metastatic prostate cancer. Current oncology reports. 2019;21:1-9.
60. Lucas Lucas C, García Zoghby L, Amo-Salas M, Soriano Castrejón ÁM, García Vicente AM. Diagnostic and therapeutic impact of PET/CT with 18F-DCFPyL versus 18F-Fluorocholine in initial staging of intermediate-/high-risk prostate cancer: a pilot study. Annals of Nuclear Medicine. 2023;37(10):551-60.
61. Долгушин М, Мещерякова Н, Оджарова А, Матвеев В, Невзоров Д, Платонова О, et al. Позитронная эмиссионная томография, совмещенная с компьютерной томографией, с 18F-ПСМА-1007 в диагностике рецидива рака предстательной железы: клиническое наблюдение. Онкоурология. 2018(3):134-8.
62. Zhao B, Dong A, Zuo C. Prostate-Specific Membrane Antigen–Avid Bone Metastases From Urothelial Carcinoma of the Bladder. Clinical Nuclear Medicine. 2022;47(10):892-4.
63. Grawe F, Blom F, Winkelmann M, Burgard C, Schmid-Tannwald C, Unterrainer LM, et al. Reliability and practicability of PSMA-RADS 1.0 for structured reporting of PSMA-PET/CT scans in prostate cancer patients. European Radiology. 2024;34(2):1157-66.
64. Werner RA, Bundschuh RA, Bundschuh L, Javadi MS, Leal JP, Higuchi T, et al. Interobserver agreement for the standardized reporting system PSMA-RADS 1.0 on 18F-DCFPyL PET/CT imaging. Journal of Nuclear Medicine. 2018;59(12):1857-64.
65. Petersen LJ, Nielsen JB, Dettmann K, Fisker RV, Haberkorn U, Stenholt L, et al. 68Ga-PSMA PET/CT for the detection of bone metastasis in recurrent prostate cancer and a PSA level< 2 ng/ml: Two case reports and a literature review. Molecular and Clinical Oncology. 2017;7(1):67-72.
66. Tatar G, Ergül N, Baloğlu MC, Arslan E, Çermik TF. 68Ga-PSMA and 68Ga-FAPI-04 PET/CT findings with 18F-FDG PET/CT in a patient with recurrent prostate cancer. Clinical Nuclear Medicine. 2023;48(3):e135-e7.
67. Alipour R, Azad A, Hofman MS. Guiding management of therapy in prostate cancer: time to switch from conventional imaging to PSMA PET? Therapeutic advances in medical oncology. 2019;11:1758835919876828.
68. Regula N, Kostaras V, Johansson S, Trampal C, Lindström E, Lubberink M, et al. Comparison of 68Ga-PSMA PET/CT with fluoride PET/CT for detection of bone metastatic disease in prostate cancer. European Journal of Hybrid Imaging. 2022;6(1):5.
69. Filippov A, Bonjoc K-JC, Chea J, Bowles N, Poku E, Chaudhry A. Role of theranostics in thoracic oncology. Journal of thoracic disease. 2020;12(9):5140.

70. Al-Ibraheem A, Hammoudeh R, Kasasbeh N, Abdlkadir AS, Juweid ME. Synchronous colorectal and prostate cancer: dual PET/CT approach for detecting and distinguishing metastatic patterns. Nuclear Medicine and Molecular Imaging. 2023;57(6):291-4.
71. Werner RA, Hartrampf PE, Fendler WP, Serfling SE, Derlin T, Higuchi T, et al. Prostate-specific membrane antigen reporting and data system version 2.0. European urology. 2023;84(5):491-502.
72. Loeff CC, van Gemert W, Privé BM, van Oort IM, Hermsen R, Somford DM, et al. [18F] PSMA-1007 PET for biochemical recurrence of prostate cancer, a comparison with [18F] Fluciclovine. EJNMMI reports. 2024;8(1):38.
73. Avul AR, Özdemir B, Altun GD. A Case Report: the Role of Prostate-Specific Membrane Antigen Labeled Theranostic Agents in the Diagnosis and Treatment of Prostate Cancer. Turkish Medical Student Journal. 2019;6(1).
74. Tuncel M, Tuncal MÇ, Telli T, Erman M. Clinical impact of PET imaging in patients with metastatic prostate cancer. Clinical nuclear medicine. 2020;45(10):757-64.
75. Jadvar H, Abreu AL, Ballas LK, Quinn DI. Oligometastatic prostate cancer: current status and future challenges. Journal of Nuclear Medicine. 2022;63(11):1628-35.
76. Pouliot F, Saad F, Rousseau E, Richard PO, Zamanian A, Probst S, et al. Intrapatient intermetastatic heterogeneity determined by triple-tracer PET imaging in mCRPC patients and correlation to survival: the 3TMPO cohort study. Journal of Nuclear Medicine. 2024;65(11):1710-7.
77. Abdel-Aty H, Hujairi N, Murray I, Yogeswaran Y, van As N, James N. The quantitative impact of prostate-specific membrane antigen (PSMA) PET/CT staging in newly diagnosed metastatic prostate cancer and treatment-decision implications. BJR| Open. 2024;6(1):tzae040.
78. Werner RA, Bundschuh RA, Bundschuh L, Javadi MS, Higuchi T, Weich A, et al. Molecular imaging reporting and data systems (MI-RADS): a generalizable framework for targeted radiotracers with theranostic implications. Annals of nuclear medicine. 2018;32:512-22.
79. Hoffman A, Amiel GE. The impact of PSMA PET/CT on modern prostate cancer management and decision making—the urological perspective. Cancers. 2023;15(13):3402.
80. Vargas-Ahumada JE, González-Rueda SD, Sinisterra-Solís FA, Casanova-Triviño P, Pitalúa-Cortés Q, Soldevilla-Gallardo I, et al. Diagnostic Performance of 99mTc-iPSMA SPECT/CT in the Initial Staging of Patients with Unfavorable Intermediate-, High-, and Very High-Risk Prostate Cancer: A Comparative Analysis with 18F-PSMA-1007 PET/CT. Cancers. 2023;15(24):5824.
